# Host relationships and biological notes of Cassidinae beetles (Coleoptera, Chrysomelidae) in Qiannan Prefecture, Guizhou, China

**DOI:** 10.3897/BDJ.12.e116267

**Published:** 2024-02-08

**Authors:** Chaokun Yang, Chengqing Liao, Jiasheng Xu, Xiaohua Dai

**Affiliations:** 1 Leafminer Group, School of Life Sciences, Gannan Normal University, Ganzhou, China Leafminer Group, School of Life Sciences, Gannan Normal University Ganzhou China; 2 National Navel-Orange Engineering Research Center, Ganzhou, China National Navel-Orange Engineering Research Center Ganzhou China

**Keywords:** faunal composition, host plant, leaf miner, insect behaviour, bipartite food web

## Abstract

The faunal composition, host relationships and biological information of the subfamily Cassidinae (Coleoptera, Chrysomelidae) remain poorly known in many Chinese regions. Based on the seven-year field survey, faunal composition and host associations of Cassidinae beetles were systematically compiled for Qiannan Buyi and Miao Autonomous Prefecture, Guizhou Province. In particular, through direct field observations, detailed biological information, such as life history and behavioural features and host plants were first recorded for 56 species of Cassidinae beetles. We have tripled the number of Cassidinae species in Qiannan. Sixty-nine species of Cassidinae beetles belonging to 17 genera and eight tribes were identified, of which 38 species are newly recorded in Guizhou and 56 are newly recorded in Qiannan. The tribes Leptispini and Notosacanthini were newly recorded in Guizhou. The genera *Thlaspidosoma* Spaeth, *Downesia* Baly, *Klitispa* Uhmann, *Platypria* Guérin-Méneville, *Leptispa* Baly and *Notosacantha* Chevrolat were recorded in Guizhou for the first time. A total of 61 species, 37 genera and 17 families of host plants were collected. Lardizabalaceae and Araliaceae were new host plant families for Cassidinae worldwide. Quantitative food web analysis indicated that Cassidinae species in Qiannan mainly feed on Poaceae, Rosaceae, Convolvulaceae and Lamiaceae. Callispini and Leptispini only feed on monocots, Aspidimorphini, Basiprionotini, Cassidini and Notosacanthini only feed on dicots, while Hispini feed on both monocots and dicots. The feeding patterns and corresponding damage marks of Cassidinae were quite diverse. In addition, the pupal mine-making behaviour of *Dactylispaexcisa* (Kraatz, 1879), *D.similis* Chen et T’an, 1985 and *D.uhmanni* Gressitt, 1950 are worth further study. Although preliminary, our field survey is an essential step in understanding Cassidinae behaviour and Cassidinae-plant interactions.

## Introduction

With more than 6,200 species, 339 genera and 43 tribes around the world, the subfamily Cassidinae Gyllenhal, 1813 sensu lato is the second richest subfamily in Chrysomelidae after Galerucinae (Chen et al. 1986, Chaboo 2007, Staines 2012, Staines 2015, Borowiec and Świętojańska 2023). There are 14 tribes, 52 genera and approximately 500 species known in China (Chen et al. 1986, Qi et al. 2008, Lee et al. 2012, Lee 2015, Staines 2015, Liao et al. 2018b, Liu et al. 2019, Borowiec and Świętojańska 2023, Yang et al. 2023). All Cassidinae are phytophagous and have formed close relationships with their host plants through long-term co-evolution (Ghate et al. 2003, Sultan et al. 2008). Host plants of the subfamily range from herbs and lianas to trees (Chen et al. 1986, Staines 2015, Liu et al. 2019, Nishida et al. 2020, Özdikmen and Coral Şahin 2021, Borowiec and Świętojańska 2023, Yang et al. 2023). Larvae of Cassidinae have various feeding habits, such as open-leaf feeding, leaf mining, stem mining, leaf-shelter scraping, leaf-tube scraping and flower scraping (Chaboo 2007). Host specialisation of Cassidinae varies considerably, mostly oligophagous, but rarely polyphagous or monophagous (Leschen and Beutel 2014).

Compared with the taxonomic records and morphological descriptions of adult beetles, biological observations and ecological notes on Cassidinae beetles are relatively few in China and there are hardly any systematic reports on the biology of Cassidinae beetles in a particular Chinese region. Amongst over 500 Cassidinae species in China, less than 10% of the species have published biological information (Lin 1978, Long et al. 2006, Yang 2006, Lee et al. 2009, Lee et al. 2012, Dai et al. 2012, Liao et al. 2014, Liao et al. 2018a, Liao et al. 2018b, Liu et al. 2018a, Liu et al. 2018b, Peng et al. 2018). However, it is important to note that the study of larval life stages can reveal more about the morphological taxonomic diversity of adults (Borowiec 2009, López-Pérez et al. 2018, Yeo et al. 2018, Chaboo et al. 2023) as larval life stages differ amongst tribes (Chaboo 2007, Świętojańska 2009, Chaboo et al. 2023). The study of larval biology helps us to obtain more detailed information on ecological, evolutionary and behavioural characteristics (Vencl et al. 2011, Vencl and Srygley 2013, Chaboo et al. 2014).

Qiannan Buyi and Miao Autonomous Prefecture (Qiannan) is located in the south-central part of Guizhou Province (Zheng et al. 2013). It has a subtropical monsoon climate, with an average annual temperature of 13.6 - 19.6℃ and an average annual precipitation of 1100 - 1400 mm (Tian et al. 2013). It is mainly characterised by typical karst peak cluster landforms, with multiple nature reserves and national wetland parks (People's Government of Qiannan Buyi and Miao Autonomous Prefecture 2023). It contains the largest primary karst forest in the world (Long et al. 2005, Liu et al. 2018).

All the insect taxa in Qiannan lack a comprehensive background survey. Only some common insect groups, such as butterflies, have been investigated in several areas of Qiannan (Cheng and Li 2002, Lan et al. 2010, Chen et al. 2014, Zhang and Xiong 2020). Cassidinae beetles have only been sporadically recorded in the literature (Zhou et al. 1987, Guo et al. 1989, Li et al. 2002), but none of them includes information on community composition, biological information and diversity patterns. Based on a seven-year field survey in Qiannan, the faunal composition and host associations of Cassidinae beetles were systematically compiled. In particular, through direct field observations, detailed biological information, such as life history and behavioural features, was first recorded for many Cassidinae species. This study may provide essential data for the conservation and utilisation of insect biodiversity resources in Qiannan.

## Materials and methods

The study was conducted in Qiannan (Fig. 1). The fieldwork was carried out in the following areas: Maolan National Nature Reserve (Maolan) (107°52′10″ – 108°05′40″ E, 25°09′20″ – 25°20′50″ N) (Fig. 2a), Doupengshan Nature Reserve (Doupengshan) (107°17′ – 107°24′ E, 26°20′ – 26°25′ N) (Fig. 2b), Yunwushan (107°03′E, 26°22′N) (Fig. 2c), Yangliu Village (107°10′E, 26°35′N) (Fig. 2d), Yanxia Nature Reserve (Yanxia) (107°15′ – 107°33′ E, 26°20′ – 26°25′ N) (Fig. 2e) and Landingshan Nature Reserve (Landingshan) (107°51′ – 107°56′ E, 25°27′ – 25°32′ N) (Fig. 2f).

Cassidinae were surveyed in 2014 (March, July and August), 2018 (July), 2019 (July and August), 2020 (January, February, May and June), 2021 (January, May, June and August), 2022 (January, May, June, July and August) and 2023 (February and June). Based on typical feeding marks on potential host leaves, Cassidinae adults or larvae were carefully checked and manually collected when walking along the surveying roads. Tree branches or whole plants harbouring Cassidinae beetles were collected and brought to the Leafminer Laboratory, School of Life Sciences, Gannan Normal University, Ganzhou, Jiangxi for rearing (Dai et al. 2012, Liao et al. 2014). The different types of behaviour of both adults and larvae were observed, recorded and photographed directly in the field or laboratory. Adults were preliminarily identified to tribe, genus and species according to literature and online resources (Chen et al. 1986, Staines 2015, Borowiec and Świętojańska 2023). The identification results were then carefully rechecked by Dr. Lukáš Sekerka (Natural History Museum, Czech Republic). Plant species were identified by Prof. Xiaoya Yu (Qiannan Normal University for Nationalities). All Cassidinae specimens were deposited at the Nanling Herbarium, Gannan Normal University (GNNU). The digital photos were taken by Chaokun Yang using the SONY A7RIV+LAOWA 25 mm photo camera.

Bipartite food-web plots were adopted to present the associations between Cassidinae and their host plants. Numeric matrices were first constructed for: (1) plant family-Cassidinae tribe, (2) plant family-Cassidinae genus, (3) plant genus-Cassidinae tribe and (4) plant genus-Cassidinae genus. The values of the plant-Cassidinae matrices were the number of Cassidinae species feeding on a particular plant family or a plant genus and the nulls in the matrices were filled with 0. The above plotting was performed using the “bipartite” package (Dormann et al. 2008, Dormann et al. 2009, Dormann 2011, Yang et al. 2023) in R 4.3.2 (R Core Team 2023) and RStudio (RStudio Team 2023).

Cassidinae classification, taxonomic names and distribution followed Chen et al. (1986), Staines (2015) and Borowiec and Świętojańska (2023). Host plant scientific names were checked on the Taxonomic Name Resolution Service (TNRS) website (https://tnrs.biendata.org/) (Boyle et al. 2021). The map was produced with QGIS 3.26.3 (QGIS Development Team 2023).

## Results

### A checklist of Cassidinae beetles and their host plants in Qiannan Prefecture

**Figure 5.**
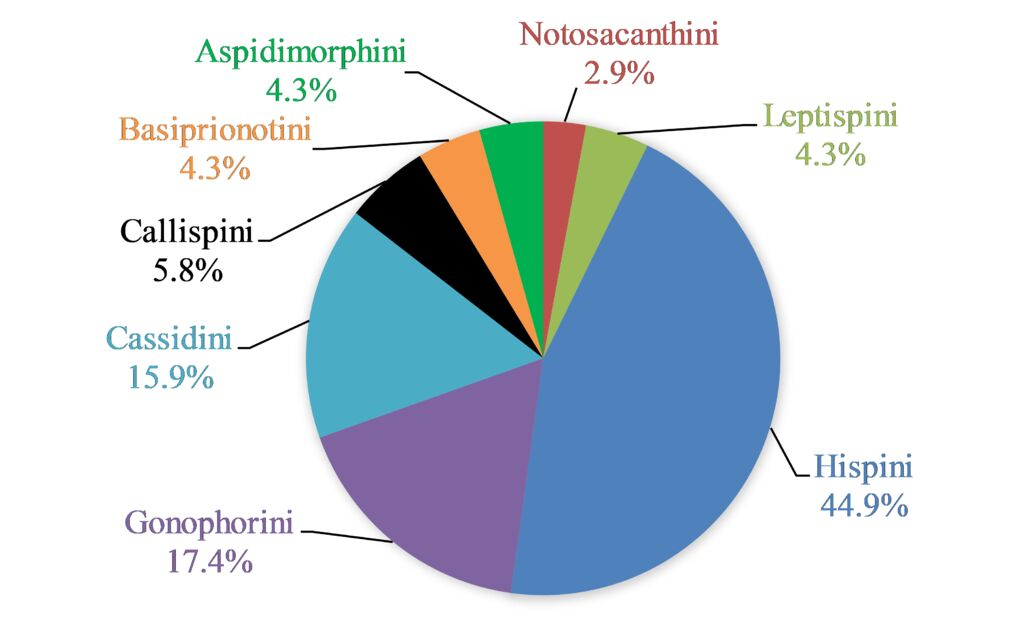
Percentage distribution of species in the eight Cassidinae tribes in Qiannan Prefecture, Guizhou, China.

As of June 2023, 2,208 individuals were collected and identified to the species level, except 109 individuals to the genus level. In total, 69 species, 17 genera and eight tribes were obtained in Qiannan (Table 1 and Suppl. material 1) and we depict the most representative species in each tribe (Figs 3, 4). Both Cassidini and Hispini have the most genera represented in our sampling, each with four genera, followed by Gonophorini, with three genera. The tribes with the fewest genera were Basiprionotini, Callispini, Leptispini and Notosacanthini, with one genus each. Hispini had the most significant number of species (31 species), accounting for 44.9% of the total, followed by Gonophorini (12 species, 17.4%) and Cassidini (11 species, 15.9%). Notosacanthini had the fewest species, with two species (Fig. 5).

The tribes Leptispini and Notosacanthini are recorded in Guizhou Province for the first time. *Thlaspidosoma* Spaeth, 1901, *Downesia* Baly, 1858, *Klitispa* Uhmann, 1939, *Platypria* Guérin-Méneville, 1840, *Leptispa* Baly, 1858 and *Notosacantha* Chevrolat, 1837 were newly-recorded genera in Guizhou. At the species level, 38 species are new records for Guizhou and 56 are new for Qiannan. Hispini has 17 species recorded for the first time in Guizhou and 27 species are newly recorded in Qiannan. Gonophorini has eleven species recorded for the first time in Guizhou and twelve species are newly recorded in Qiannan. Cassidini, Callispini and Leptispini each have three species recorded for the first time in Guizhou. Notosacanthini has two species recorded for the first time in Guizhou.

### Host plant relationships of Cassidinae beetles in Qiannan Prefecture

A total of 61 species, 37 genera and 17 families of host plants were collected in Qiannan (Suppl. material 2). Amongst them, Araliaceae and Lardizabalaceae are new host family records for Cassidinae worldwide. Rosaceae had the most significant host species, with 27.9% of the total (n = 17), followed by Poaceae (24.6%, n = 15). Most Cassidinae species were collected from Poaceae, Rosaceae and Convolvulaceae.

According to the quantitative food-web plots (Fig. 6), Poaceae was fed on by the most Cassidinae species, consisting of four tribes and eight genera, followed by Lamiaceae feeding by three Cassidinae tribes and three genera (Fig. 6). At the Cassidinae tribe level, Hispini fed on the most significant number of host plant families and genera, with eight families (Fig. 6a) and 19 genera (Fig. 6c), followed by Cassidini, with six host plant families and twevel genera. Aspidimorphini, Callispini and Leptispini each feeding on only a single host plant family (Fig. 6a). At the genus level, *Dactylispa* Weise had the most significant number of host plant families and genera, with seven families (Fig. 6b) and 18 genera (Fig. 6d), followed by *Cassida* Linnaeus, with four host plant families (Fig. 6b) and nine genera (Fig. 6d). *Klitispa* Uhmann, *Hispellinus* Weise, *Rhadinosa* Weise and *Thlaspida* Weise each fed on only a single host plant family and genus.

Amongst the 66 species with host records, most species (38 species) feed on dicotyledons plants. Callispini, Gonophorini and Leptispini all feed only on monocotyledons (traditional Hispinae), while Hispini can feed on both monocotyledons and dicotyledons. Aspidimorphini, Cassidini, Basiprionotini and Notosacanthini (traditional Cassidinae) all feed only on dicotyledons.

### Biological notes of some Cassidinae beetles in Qiannan Prefecture

#### Tribe Aspidimorphini

All Aspidimorphini species we collected feed on Convolvulaceae. The adults of *Aspidimorpha* (*s. str.*) *furcata* (Thunberg, 1789) appear in early May with adults feeding on different Convolvulaceae species. On the host *Calystegiasepium* (L.) R.Br. (Fig. 7a), both larvae and adults usually feed on the epidermis of young leaves. Larvae leave irregularly-shaped feeding scars when feeding on the lower side of the leaves and sometimes on the upper side, but the resulting feeding scars do not penetrate the leaf. Larvae carry faecal shields composed of exuvia and faeces, which are attached to the supra-anal processes (Fig. 7b). Adults usually feed on the lower side of the leaves (Fig. 7c, d) and leave large, irregular oval holes, some penetrating the leaf margin (Fig. 7d).

Adults of Laccoptera (Laccopteroidea) nepalensis Boheman, 1855 also appear in early May to feed on different Convolvulaceae species. On the host *Ipomoeapurpurea* (L.) Roth (Fig. 8a), both larvae and adults feed on the epidermis of young leaves. Larvae leave irregularly-shaped feeding scars on the upper side of the leaf and the lower epidermis commonly remains intact (Fig. 8b); however, adults always leave oval holes (Fig. 8c). The larvae carry a faecal shield composed of exuvia and faeces, which are attached to the supra-anal processes. (Fig. 8b). On sunny days, adults were very active, constantly flying around the host plants. During the morning, adults can often be seen copulating on the leaf surface (Fig. 8d). The females lay their eggs in leaf epidermal depressions (usually in the veins).

#### Tribe Basiprionotini

*Basiprionotapudica* (Spaeth, 1925) is one of the species with the most complete life stages that we observed in Qiannan. Adults usually appear in mid-April. Both larvae and adults feed on the epidermis of young leaves of *Premnamicrophylla* Turcz. (Lamiaceae) (Fig. 9a). Adults always lay their eggs, which are creamy-yellow and surrounded by a transparent white membrane, in a depression on the upper side of the leaf (usually on a leaf vein) (Fig. 9b). Larva feed on the plant by dragging their mandibles across the surface of the leaf and leaving irregularly-shaped feeding scars on the upper side of the leaf (Fig. 9c). The feeding scar does not penetrate the leaf. The larva is covered dorsally with an exuvial stack of excreted faecal filaments. Larvae carry faecal shields composed of exuvia and faeces, which are attached to the supra-anal processes. Larvae usually pupate on the upper side of the leaf (Fig. 9d). Adult feeding usually leaves large, irregular oval holes on leaves, some penetrating the leaf margin (Fig. 9e). Adults usually copulate on the leaf surface (Fig. 9f).

#### Tribe Callispini

*Callispabowringii* Baly, 1858 feeds on different Poaceae species. Larvae appear in mid-April. Both larvae and adults are open-leaf feeders, which make oblong channels on the leaves (Fig. 10b, c, d). Larva feed on the lower side of the leaf. The larva deposits its faecal matter on its supra-anal processes, but does not form a faecal shield (Fig. 10b). Adults usually feed on the lower side of the leaf (Fig. 10c), sometimes on the upper side of the leaf (Fig. 10d). Adults overwinter on the lower leaf side of *Indocalamustessellatus* (Munro) Keng f. (Poaceae) or *Pleioblastusamarus* (Keng) Keng f. (Poaceae)

#### Tribe Cassidini

Both larvae and adults of Cassidini are open-leaf feeders. Adults of *Glyphocassisspilota* (Gorham, 1885) appear in early May. Both larvae and adults feed on young leaves of *Calystegiasepium* (L.) R.Br. (Fig. 11a). The female lays her egg individually on the young leaf surface (Fig. 11b①). Larvae prefer to feed on the lower side of young leaves and make irregularly-shaped feeding scars (Fig. 11b②), usually not penetrating the leaf. Sometimes the larvae crawl to the upper side of the leaf. The larvae carry faecal shields composed of exuvia and faeces, which are attached to the supra-anal processes. (Fig. 11c). Larvae usually pupate on the lower side of the leaf. Adults feed on the leaf lower side and usually leave large, irregular oval holes, some penetrating the leaf margin (Fig. 11d).

#### Tribe Gonophorini

In our field observations in Qiannan, all the larvae of Gonophorini were leaf miners. Both larvae and adults of *Agonitachinensis* (Weise, 1922) feed on different Poaceae species. Larvae appear in late May. Larvae mine on young leaves of *Pleioblastusamarus* (Keng) Keng f. and *Phyllostachysmannii* Gamble (Fig. 12a), which would form a large white blotch mine (Fig. 12b). Adults usually feed on the upper side of the leaf and produce elongated feeding channels (Fig. 12c), sometimes feeding on the lower side of the leaf. According to our observations, adults overwinter in groups on the leaf surface of *Pleioblastusamarus* (Poaceae) (Fig. 12d).

The biology of the genus *Downesia* is poorly known. Based on our observations, the larvae of *Downesiastrandi* Uhmann, 1943 generally appear in mid-April. Adults can usually be observed copulating in the rolled leaves (Fig. 13a). Females lay their eggs in the rolled young leaves. The larvae mine in the young leaves. More than two larvae generally mine simultaneously in a communal mine (Fig. 13b). The larval mines are large and irregular, sometimes extending to the entire leaf. Adults also feed on the rolled leaf surface and produce elongated feeding channels (Fig. 13c, d). Adults overwinter in groups in the rolled dead leaves of *Indocalamustessellatus* (Munro) Keng f. (Poaceae).

#### Tribe Hispini

Larvae of Hispini are leaf miners. Hispini uses the highest number of host families, including both monocotyledons and dicotyledons. In our field observations in Qiannan, some species of *Dactylispa* mature larvae can build one new pupal mine for pupation (e.g. *Dactylispaexcisa* Chen et T’an, *D.similis* Chen et T’an and *D.uhmanni* Gressitt); however, this phenomenon only occurs in Rosaceae.

Adults of *D.similis* generally appear in mid-May. Larvae mine in young leaves of *Rosacymosa* Tratt. (Rosaceae) (Fig. 14a), starting at the tip of the young leaf, then feed along one side of the leaf and form a large blotch mine (Fig. 14b). Mature larvae can build one new mine for pupation. Generally, the area selected for pupation is close to the leaf's mid-rib and sometimes on the mid-rib. The pupal mine looks like a U-shaped pocket on the upper side of the leaf. The pupa directs its head towards the pupal mine opening (Fig. 14c). Adults feed on the upper side of leaves and leave elongated feeding scrapings (Fig. 14d). When adults are not feeding, they crawl to the lower side of the leaf surface to rest.

*Dactylispagressitti* Uhmann, 1954 is an interesting species. To our knowledge, *D.gressitti* is known only from Fujian Province (e.g. Guadun) and no-one has collected or reported fresh specimens since the original description of the species. The larvae mine in the young leaf of *Hederanepalensis* K.Koch (Araliaceae) (Fig. 15a) in late May. The larvae feed along the sides of the main leaf veins, forming a large blotch mine (Fig. 15b) and finally pupate at the leaf veins (Fig. 15c). Adults usually feed on the lower side of the leaf, sometimes on the upper side of the leaf, leaving slightly elongated feeding scrapings, usually without penetrating the dorsal epidermal cuticle (Fig. 15d). Adults also copulate and rest on the lower side of the leaf (Fig. 15e). Adults overwinter in groups on the lower side of the leaf surface.

#### Tribe Leptispini

Very little biological information is known about Leptispini in China. Larvae of *Leptispapici* Uhmann, 1958 appear in mid-May. Adults feed on the leaves of *Bambusablumeana* Schult.f. (Poaceae) (Fig. 16a). According to our field observations, both larvae and adults live on rolled young leaves. Several larvae inside a leaf roll together (Fig. 16b). Pupae are also found in the leaf roll (Fig. 16c). Adults feed inside the leaf roll (Fig. 16d), always in groups (Fig. 16e). Both adults and larvae are leaf scrapers, making elongated feeding scars on the leaf upper surface.

## Discussion

Six tribes, 13 genera and 54 species have been reported in Guizhou Province (Chen et al. 1986, Guo et al. 1989, Hua 2002, Yang et al. 2005, Jin et al. 2006, Borowiec 2009, Li 2011, Staines 2015, Borowiec and Świętojańska 2023). However, Cassidinae beetle richness in Guizhou Province remains poorly known compared to neighbouring provinces, such as Yunnan and Guangxi, especially for endemic species. In this study, 69 Cassidinae species were gathered in Qiannan, with the majority collected in previously unrecorded areas, of which 38 species are new records for Guizhou. In our previous studies, we found 27 newly-recorded species in Jiangxi Province (Liu et al. 2019) and 33 newly-recorded species in Guangxi Zhuang Autonomous Region, China (Yang et al. 2023). The above large numbers of new records indicate that Cassidinae fauna in many Chinese regions might still be underestimated.

The frequency distributions of host plants for different Cassidinae tribes in Qiannan are quite uneven, with some plants being used much more than others. The highest number of Cassidinae species was collected from Poaceae, with 27 species from four tribes, which is consistent with previous reports from Longnan County and southern Guangxi (Liu et al. 2019, Yang et al. 2023). Such plant utilisation patterns may be explained by the plant apparency hypothesis, i.e. dominant plant groups could suffer more insect herbivores (Dai et al. 2014, Dai et al. 2017, Liu et al. 2019). In our observation in Qiannan, many Cassidinae species were monophagous (restricted to one plant species), such as *Downesiavandykei* Gressitt and *Leptispapici* Uhmann. Some Cassidinae species show limited oligophagous habits, such as *Agonitachinensis*, *Cassidaversicolor* and *Laccopteranepalensis*. *Agonitachinensis* feed on different genera of the family Poaceae, *C.versicolor* on different genera of Rosaceae and *Laccopteranepalensis* on different genera of Convolvulaceae. This study supplemented novel host plant information for many Cassidinae species, which could assist our future field survey. Information such as host plant apparency, host plant composition and host plant range may help us to save time and resources for future Cassidinae collection activities (Fernandes and Linzmeier 2012).

Based on our field observations in Qiannan, some Cassidinae larvae began to emerge in mid-April, but most larvae appeared in May. Both larvae and adults of the tribes Aspidimorphini, Basiprionotini, Cassidini and Callispini are open-leaf feeders. However, many Aspidimorphini, Basiprionotini and Cassidini larvae usually feed on the upper side of the leaves and few feed on the lower side of the leaves. The larvae of Aspidimorphini, Basiprionotini and Cassidini could construct faecal shields using cast skins and faecal strands, which are often considered protection against predators (Eisner et al. 1967, Olmstead and Denno 1993, Gómez et al. 1999, Vencl et al. 2005, Vencl et al. 2009, Vencl and Srygley 2013). Larvae of Callispini do not have dung camouflage on the dorsal side and the larvae prefer to feed on the lower leaves. This habit is consistent with *Callispatsoui* Lee, Świętojańska, & Staines and *C.keram* Shameem & Prathapan (Lee et al. 2012, Shameem and Prathapan 2013), which may also be a defensive strategy against predators. Larvae of Gonophorini, Hispini and Notosacanthini are leaf miners. Larvae and adults of Gonophorini feed only on monocotyledons. Larvae and adults of Notosacanthini feed only on dicotyledons. Larvae and adults of Hispini feed on both monocotyledons and dicotyledons. *Dactylispaexcisa*, *D.similis* and *D.uhmanni* make pupal chambers in the leaves, which are interesting and worth studying further in our future work. Pupal chambers have been reported in some dicot-feeding Hispini species, such as *Platypriaerinaceus* (Fabricius, 1801), *P.hystrix* (Fabricius, 1798) (Ranade et al. 2021), *P.melli* Uhmann, 1954 (Liao et al. 2014) and *Cassidisparelicta* Medvedev, 1957 (Liao et al. 2018b). In addition, pupal chambers are also found in other Cassidinae tribes, such as *Notosacanthadorsalis* (Waterhouse, 1877) (Monteith et al. 2021) and *Notosacanthavicaria* (Spaeth, 1913) (Nilesh et al. 2000) in Notosacanthini. *Oncocephalapromontorii* Péringuey, 1898 (Chaboo et al. 2010), *Prionispahoujayi* Lee, Swietojanska & Staines, 2009 (Lee et al. 2009) and *P.champaka* Maulik, 1919 (Liao et al. 2018a) in Oncocephalini. It has also been reported that other Leptispini larvae (e.g. *Leptispa.godwini* Baly, 1869 and *L.pygmaea* Baly, 1858) have grouping and leaf roll-feeding behaviour (Liao et al. 1981, Prathapan et al. 2009). Such diverse life histories and behaviour may be the most likely drivers for the extraordinary morphological diversity in Cassidinae, especially in larvae (Chaboo 2007).

In this study, we have tripled the number of Cassidinae species in Qiannan. Moreover, we have provided new host records and novel biological notes for many Cassidinae beetles. Although preliminary, our field survey is an essential step in understanding Cassidinae behaviour and Cassidinae-plant interactions.

## Supplementary Material

4FCACEFA-7D8A-5A2A-BC1E-1595ACD6B351 10.3897/BDJ.12.e116267.suppl1 Supplementary material 1 Cassidinae beetles and their confirmed host plants in Qiannan Prefecture, Guizhou, China Data type Checklist, taxonomic status, insect-plant associations. Brief description All identified Cassidinae beetles and their confirmed host plants. File: oo_961949.xlsx https://binary.pensoft.net/file/961949 Chaokun Yang, Chengqing Liao, Jiasheng Xu, Xiaohua Dai

B9F3A1FE-7010-56F8-81B0-0EE3F7D1700A 10.3897/BDJ.12.e116267.suppl2 Supplementary material 2 Host plants and their corresponding Cassidinae beetles in Qiannan Prefecture, Guizhou, China Data type Checklist, taxonomic status, insect-plant associations. Brief description Associations between host plants and their corresponding Cassidinae beetles. File: oo_962367.xlsx https://binary.pensoft.net/file/962367 Chaokun Yang, Chengqing Liao, Jiasheng Xu, Xiaohua Dai

## Figures and Tables

**Figure 1a.**
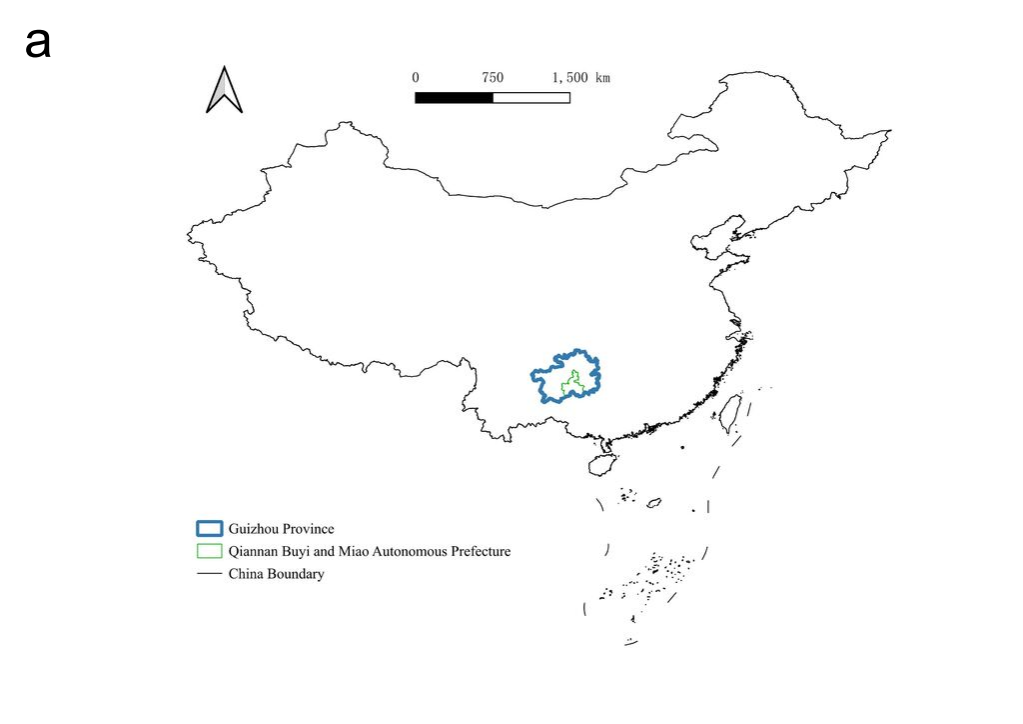
Qiannan and Guizhou in China;

**Figure 1b.**
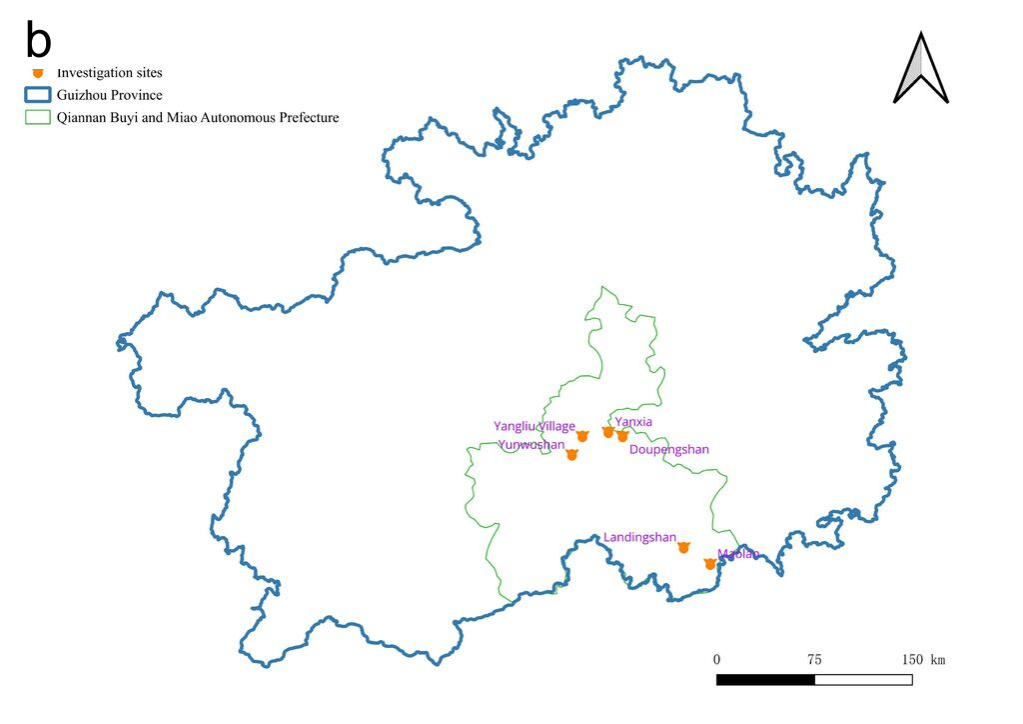
Investigation sites in Qiannan.

**Figure 2a.**
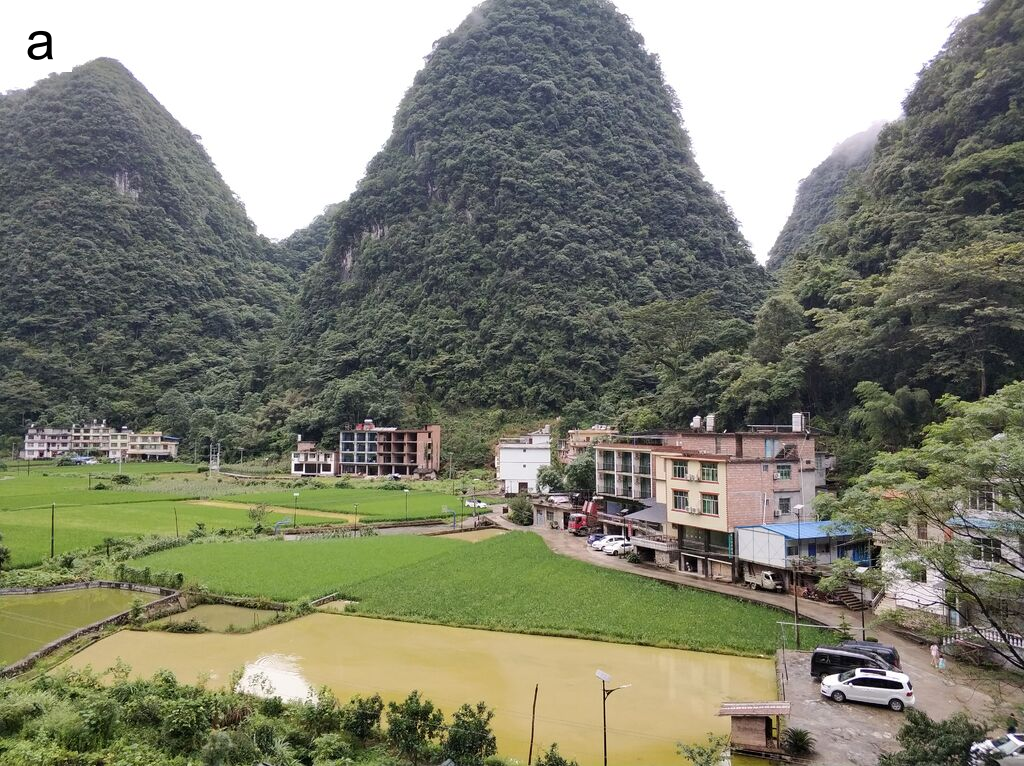
Maolan (Photo by Chaokun Yang);

**Figure 2b.**
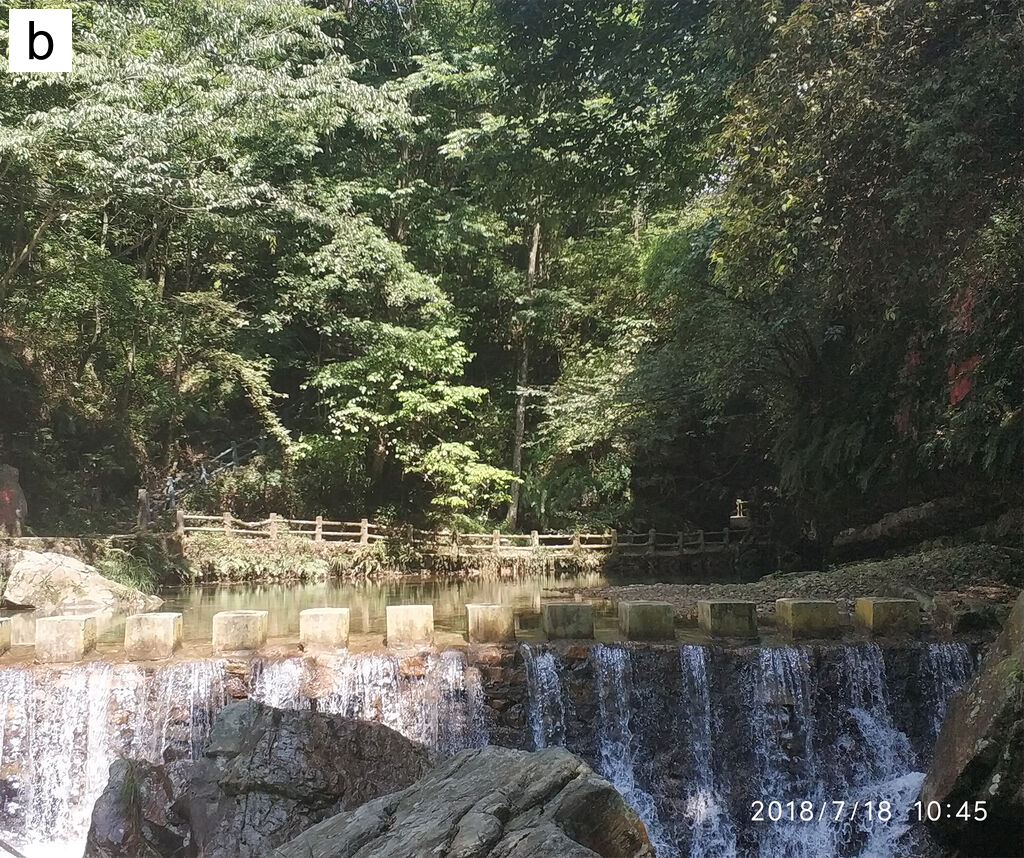
Doupengshan (Photo by Chaokun Yang);

**Figure 2c.**
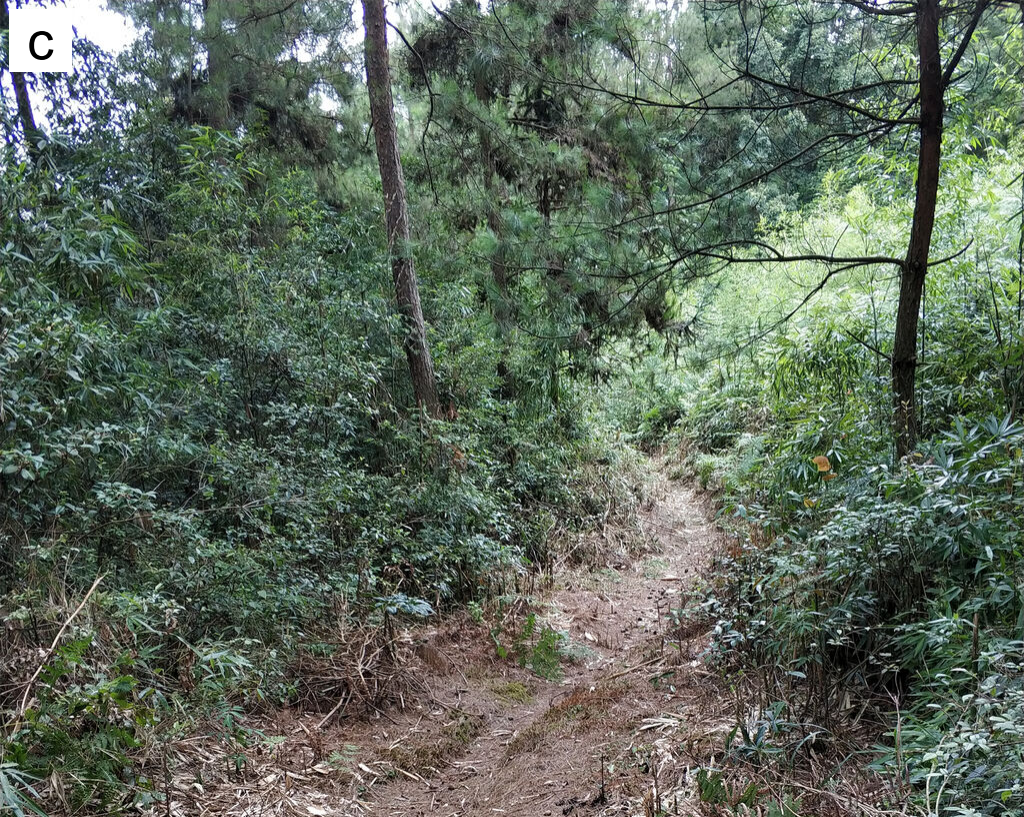
Yunwushan (Photo by Chaokun Yang);

**Figure 2d.**
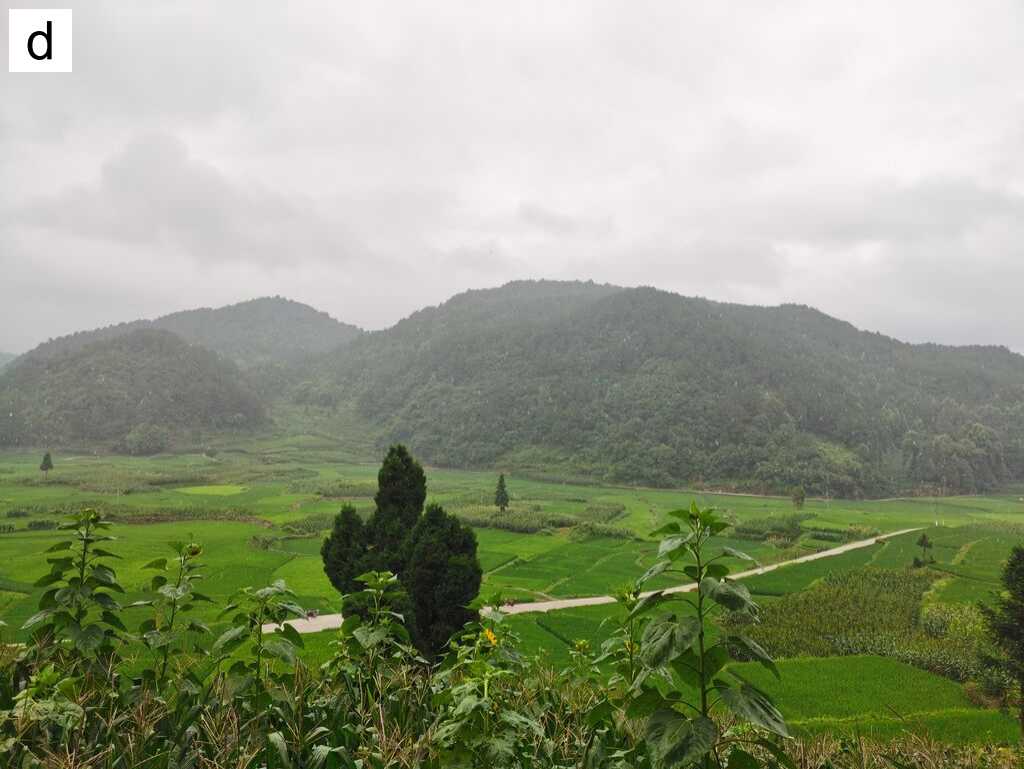
Yangliu Village (Photo by Chaokun Yang);

**Figure 2e.**
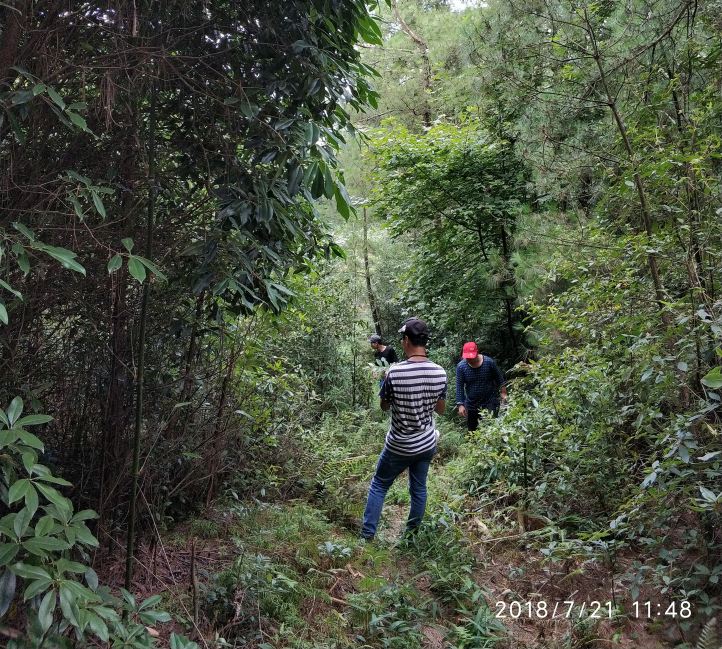
Yanxia (Photo by Chaokun Yang);

**Figure 2f.**
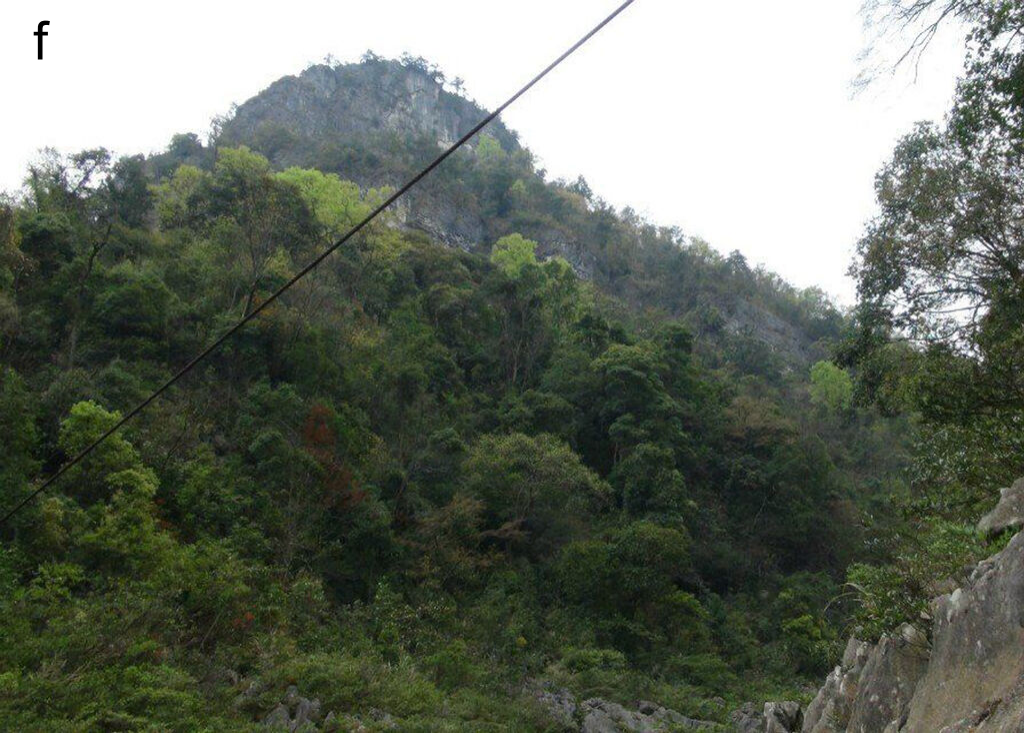
Landingshan (Photo by Xiaohua Dai).

**Figure 3a.**
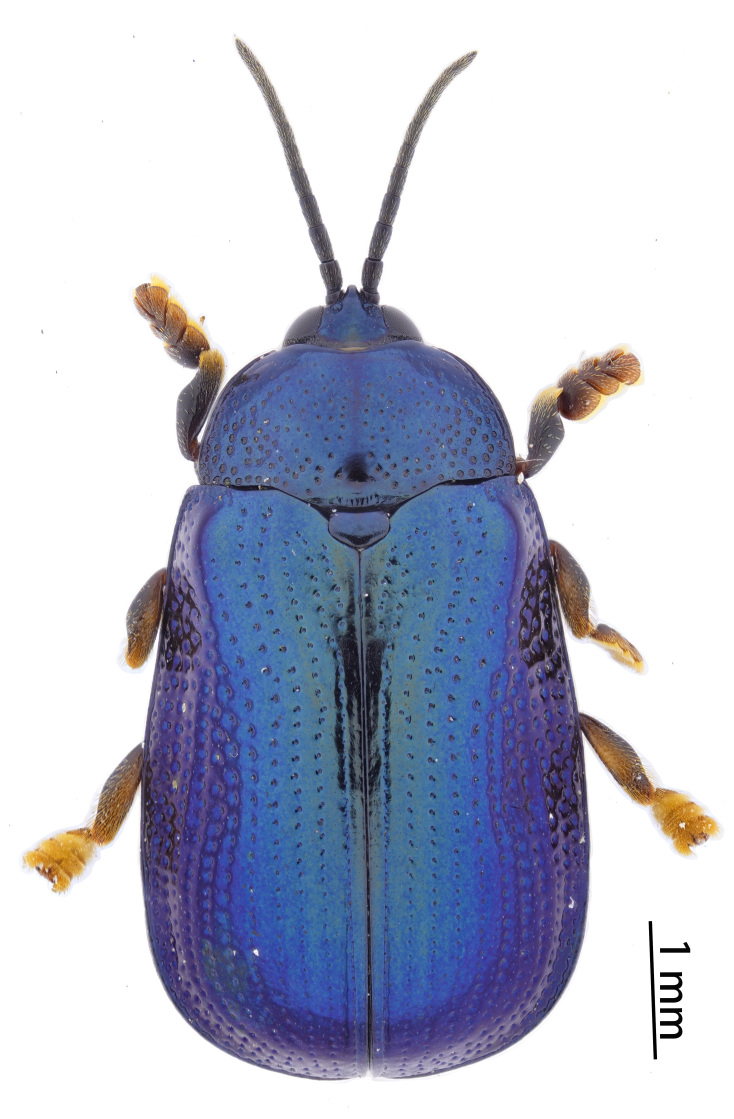
*Callispabowringii* Baly, 1858, Callispini;

**Figure 3b.**
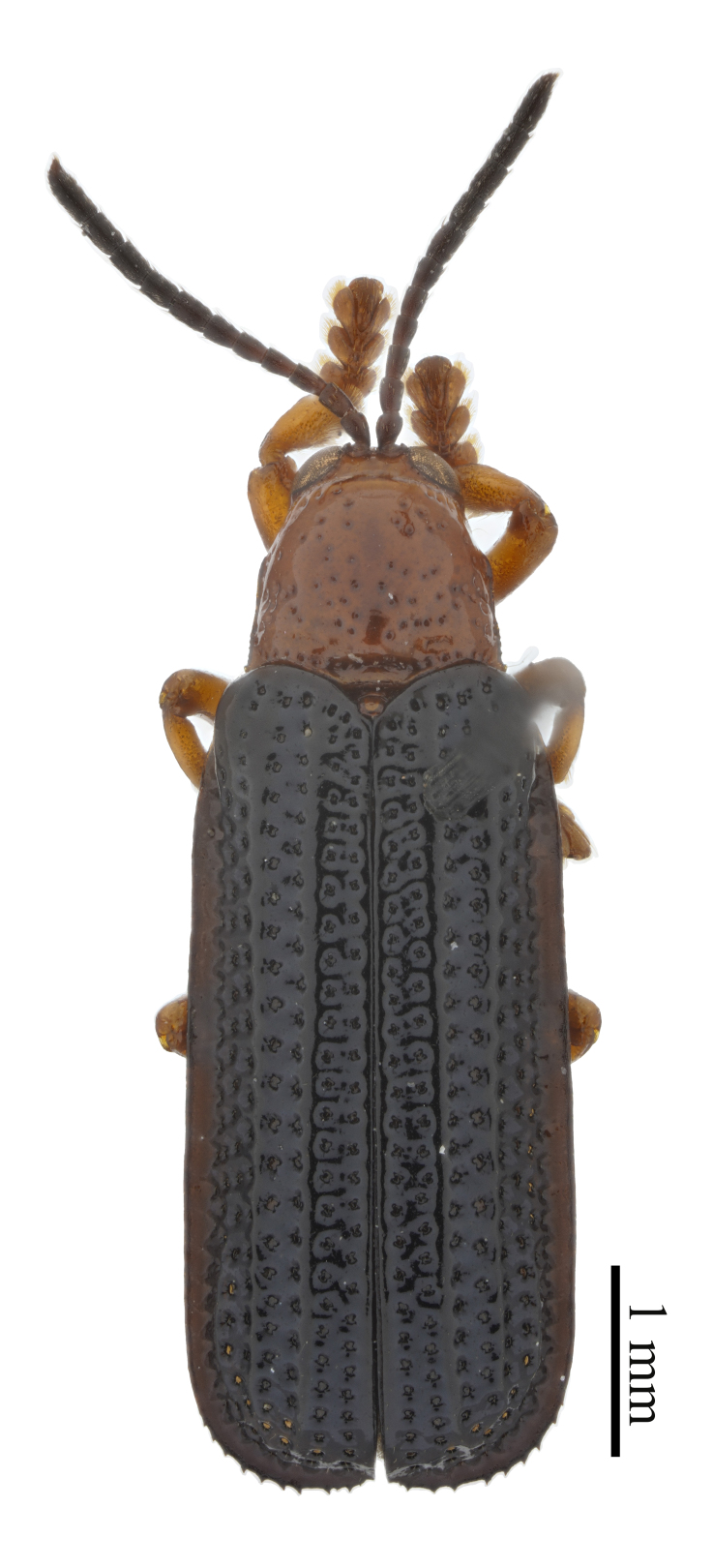
*Agonitachinensis* (Weise, 1922), Gonophorini;

**Figure 3c.**
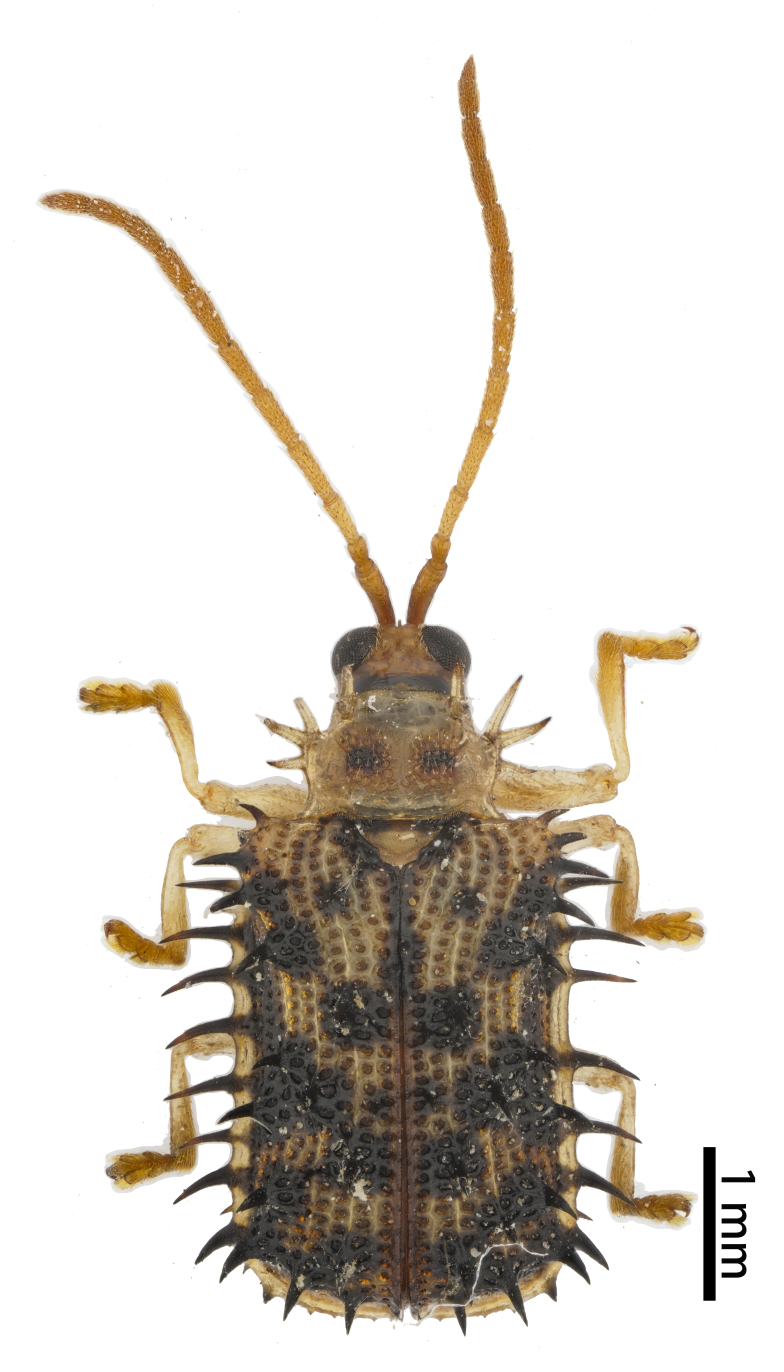
*Dactylispagressitti* Uhmann, 1954, Hispini;

**Figure 3d.**
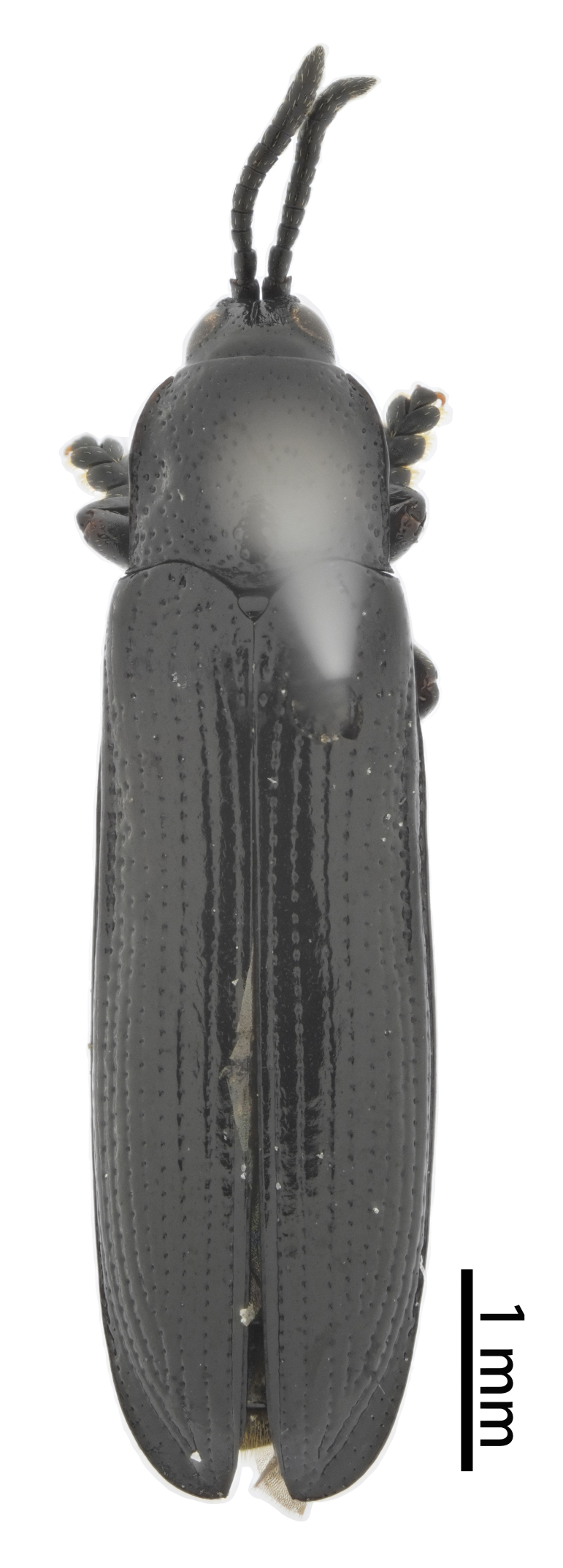
*Leptispagodwini* Baly, 1869, Leptispini.

**Figure 4a.**
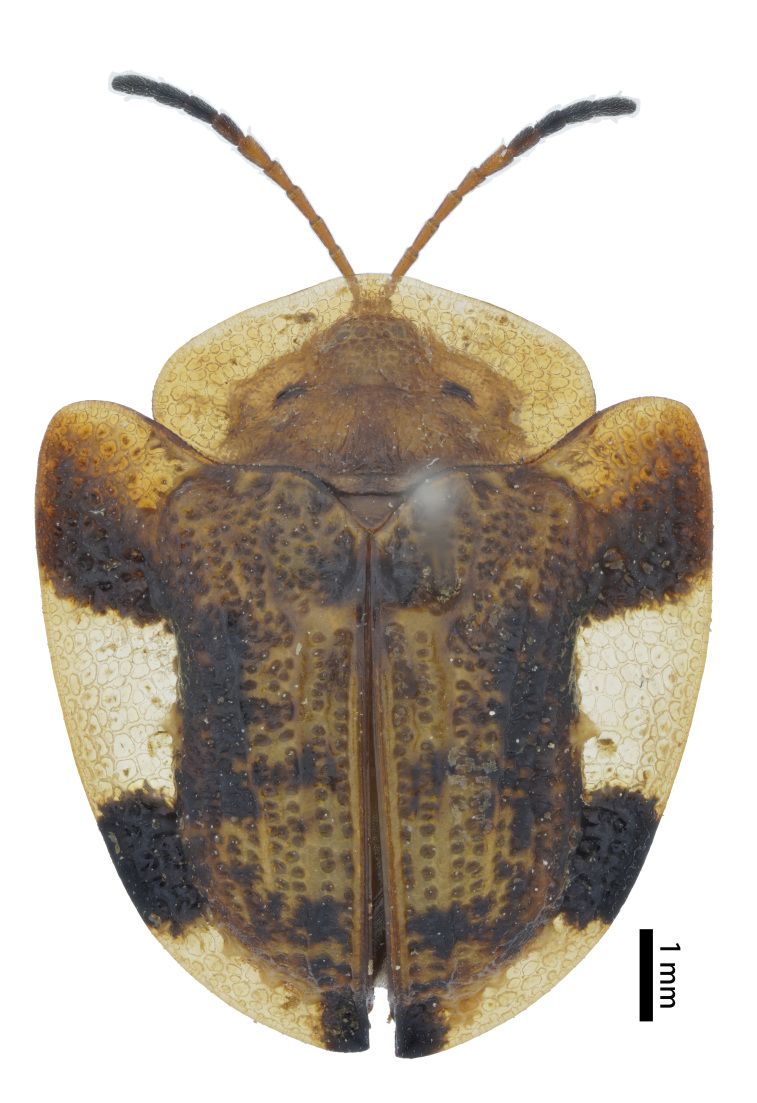
*Laccopteranepalensis* Boheman, 1855, Aspidimorphini;

**Figure 4b.**
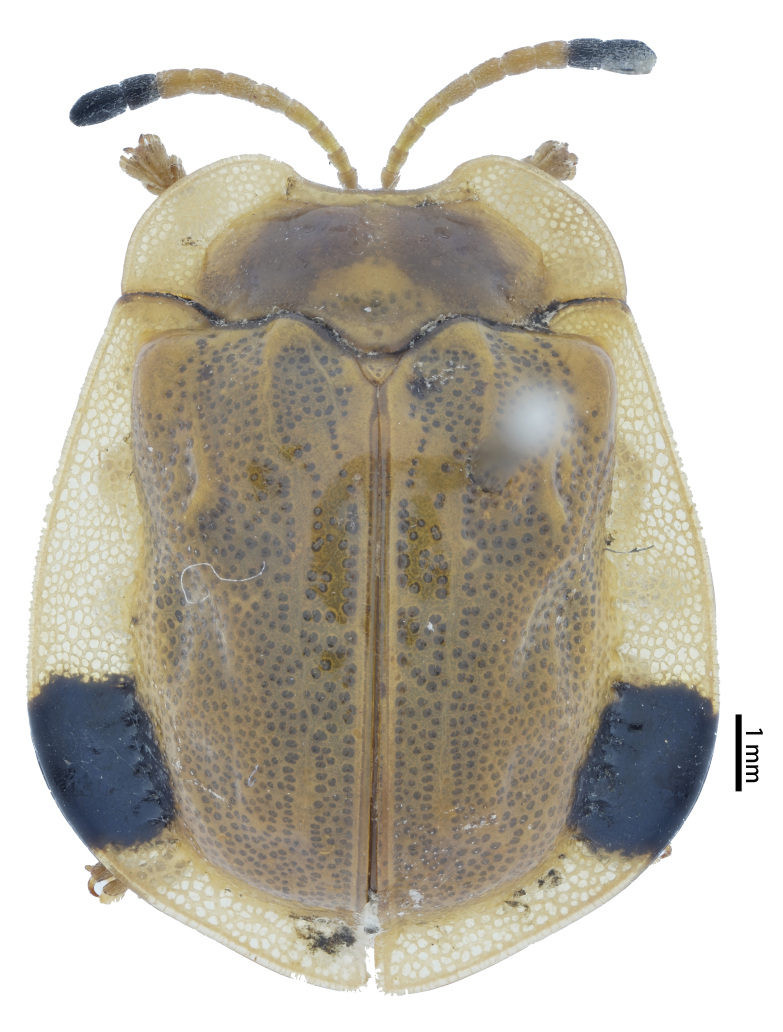
*Basiprionotapudica* (Spaeth, 1925), Basiprionotini;

**Figure 4c.**
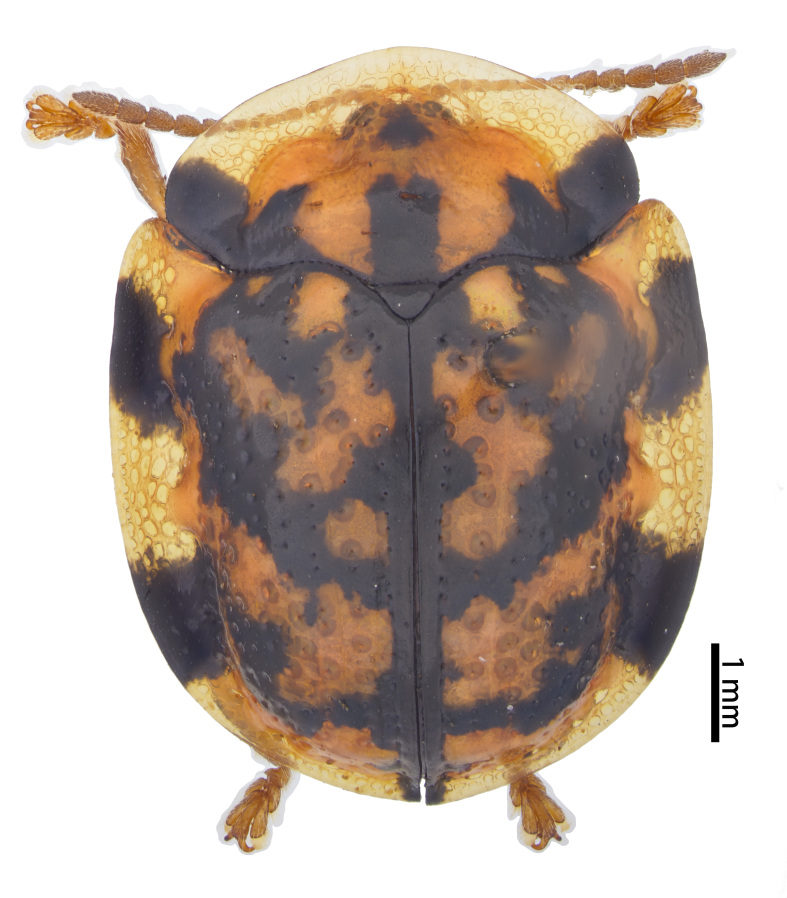
*Glyphocassisspilota* (Gorham, 1885), Cassidini;

**Figure 4d.**
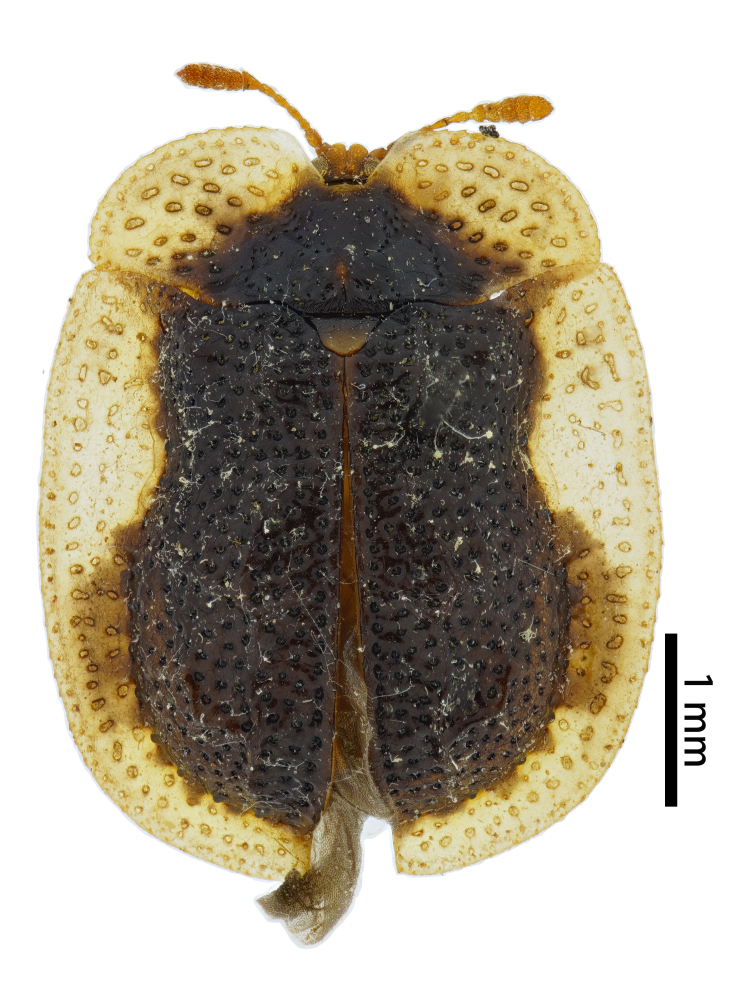
*Notosacanthaginpinensis* Chen et Zia, 1961, Notosacanthini.

**Figure 6a.**
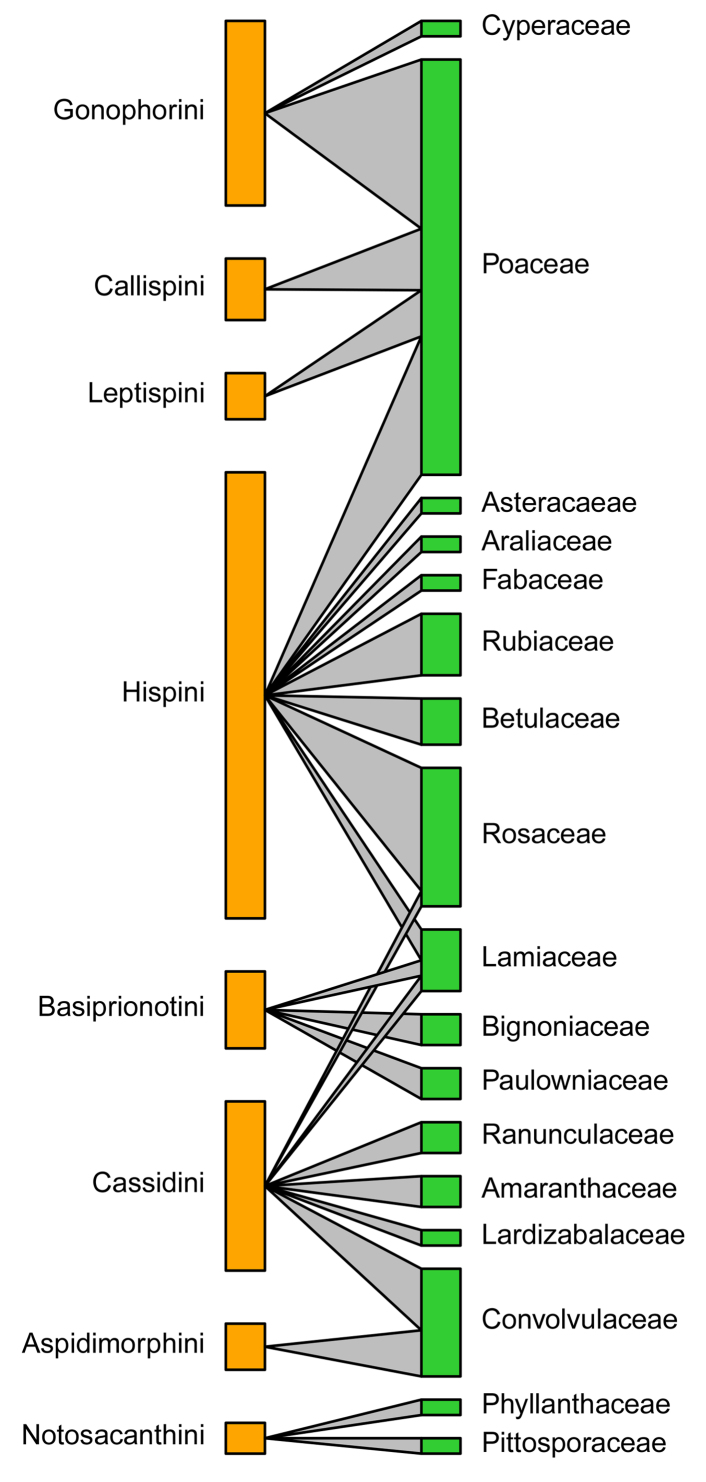
Quantitative food web amongst 17 host plant families (green) and eight Cassidinae tribes (orange);

**Figure 6b.**
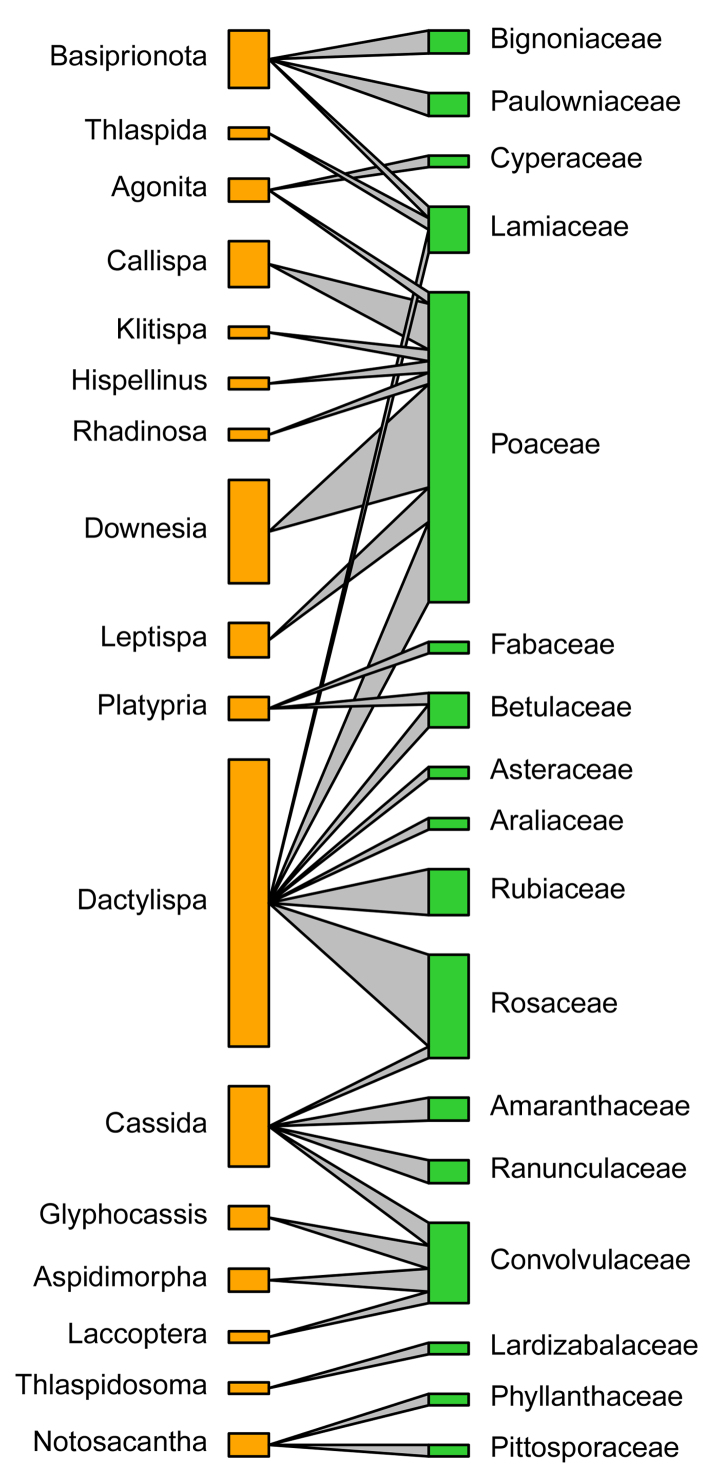
Quantitative food web amongst 17 host plant families (green) and 17 Cassidinae genera (orange);

**Figure 6c.**
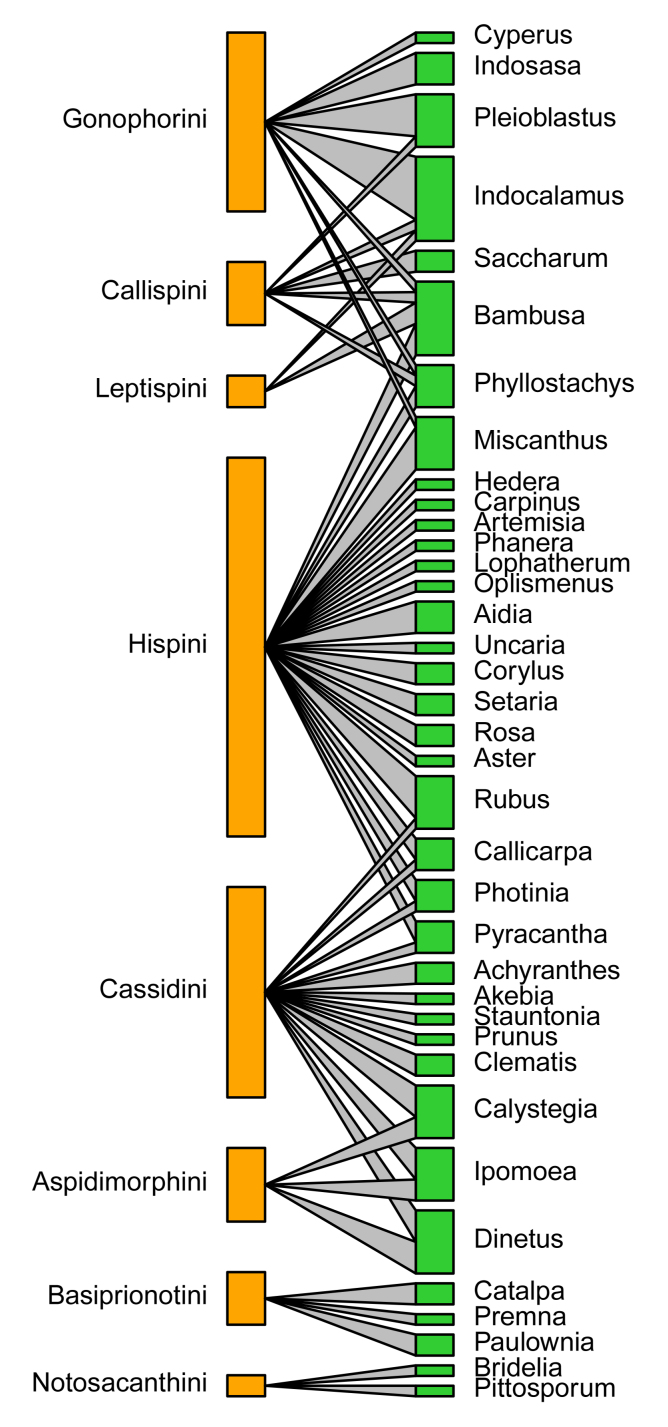
Quantitative food web amongst 37 host plant genera (green) and eight Cassidinae tribes (orange);

**Figure 6d.**
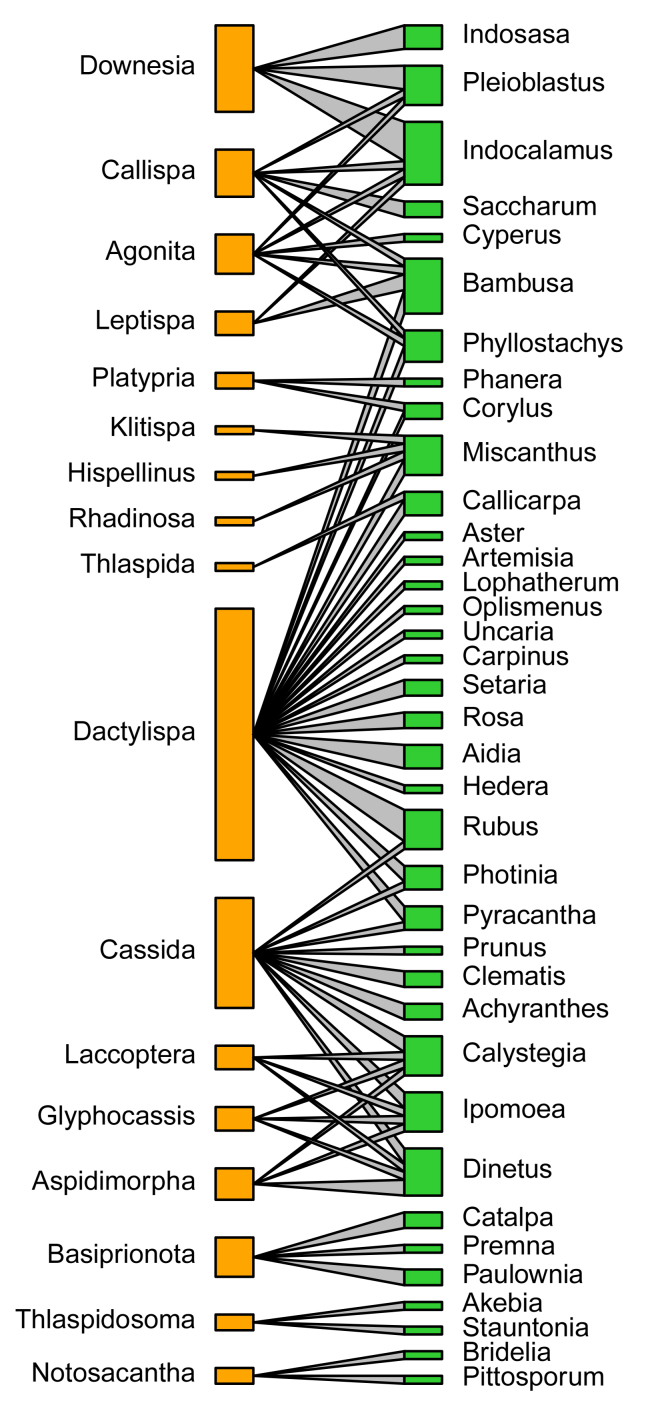
Quantitative food web amongst 37 host plant genera (green) and 17 Cassidinae genera (orange).

**Figure 7a.**
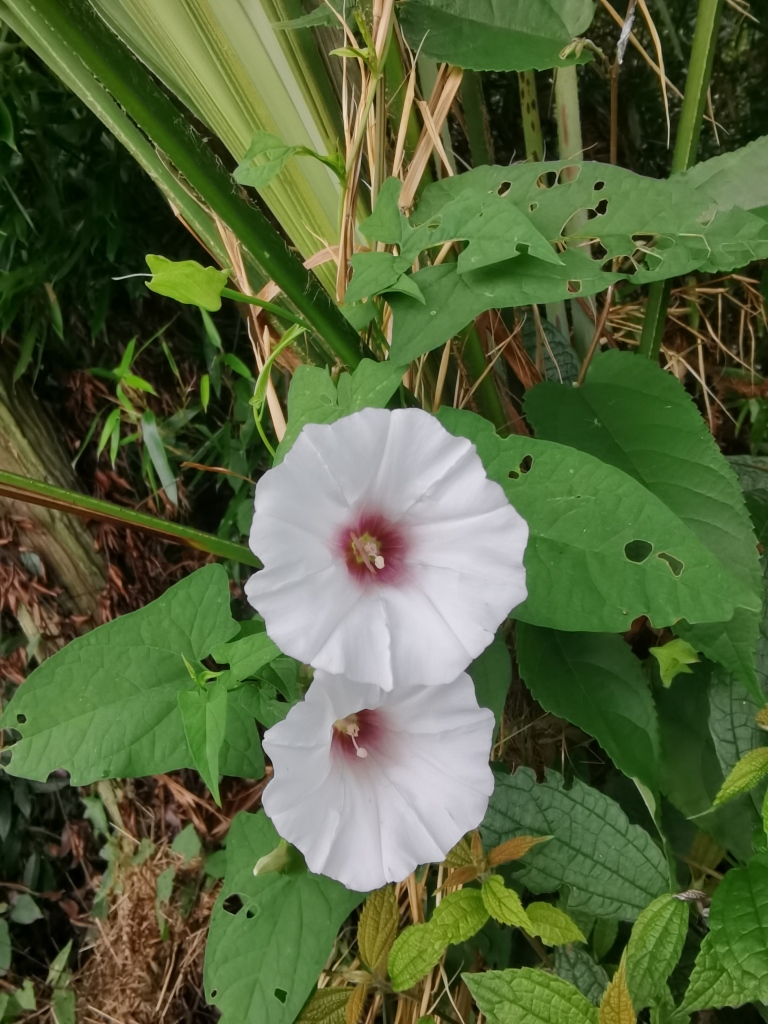
*Calystegiasepium* (L.) R.Br. (Convolvulaceae), the host plant for *A.furcata*;

**Figure 7b.**
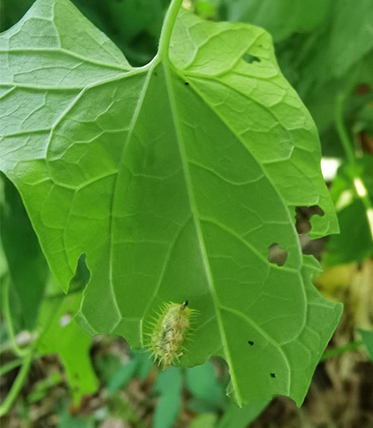
Larva;

**Figure 7c.**
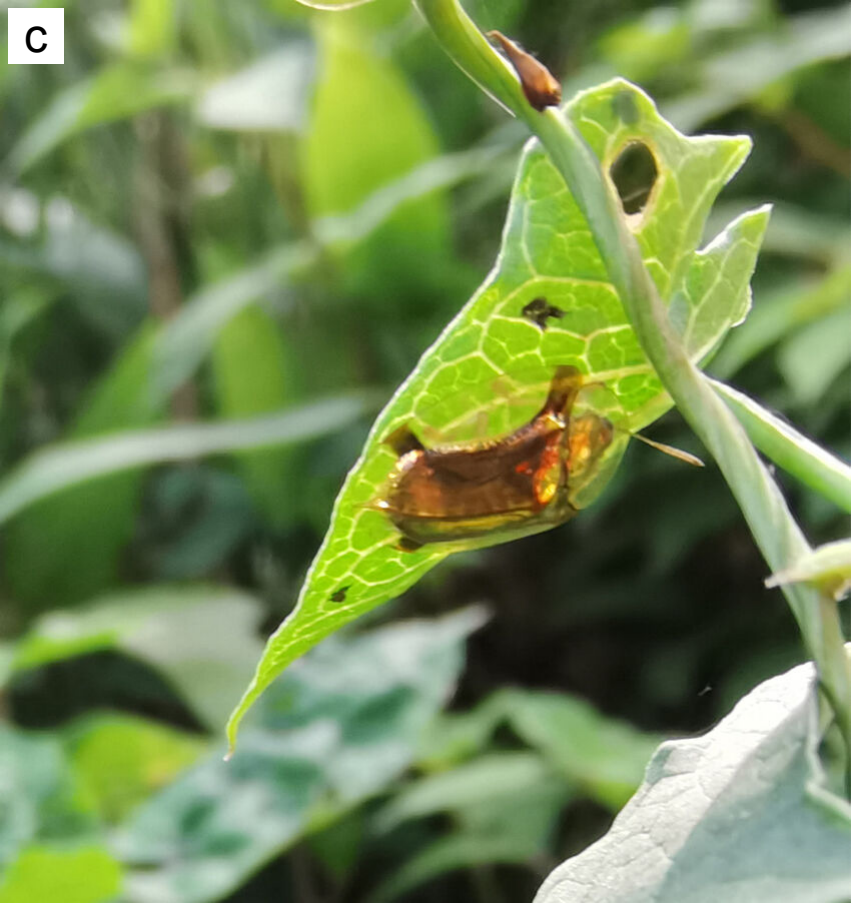
Adult;

**Figure 7d.**
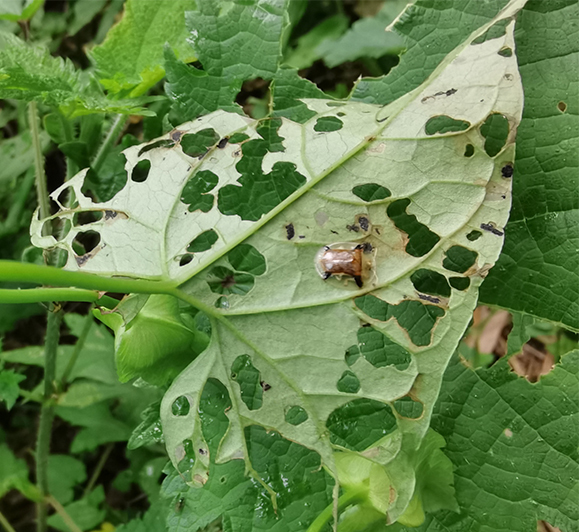
Adult and its feeding pattern.

**Figure 8a.**
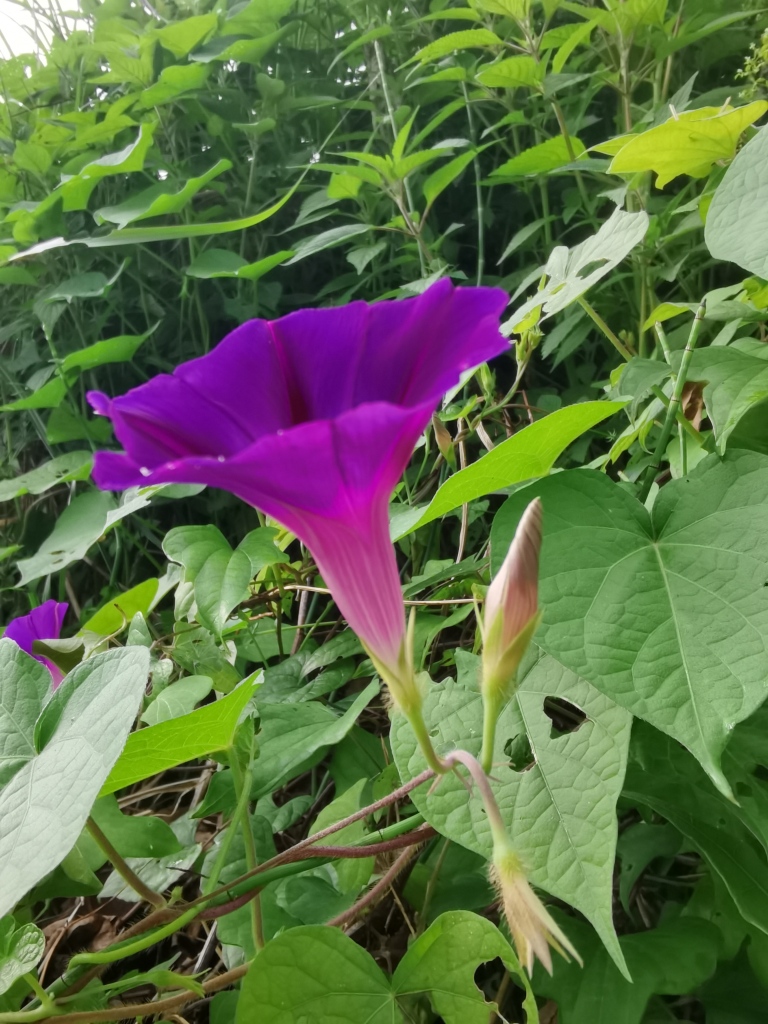
*Ipomoeapurpurea* (L.) Roth (Convolvulaceae), the host plant for *Laccopteranepalensis*;

**Figure 8b.**
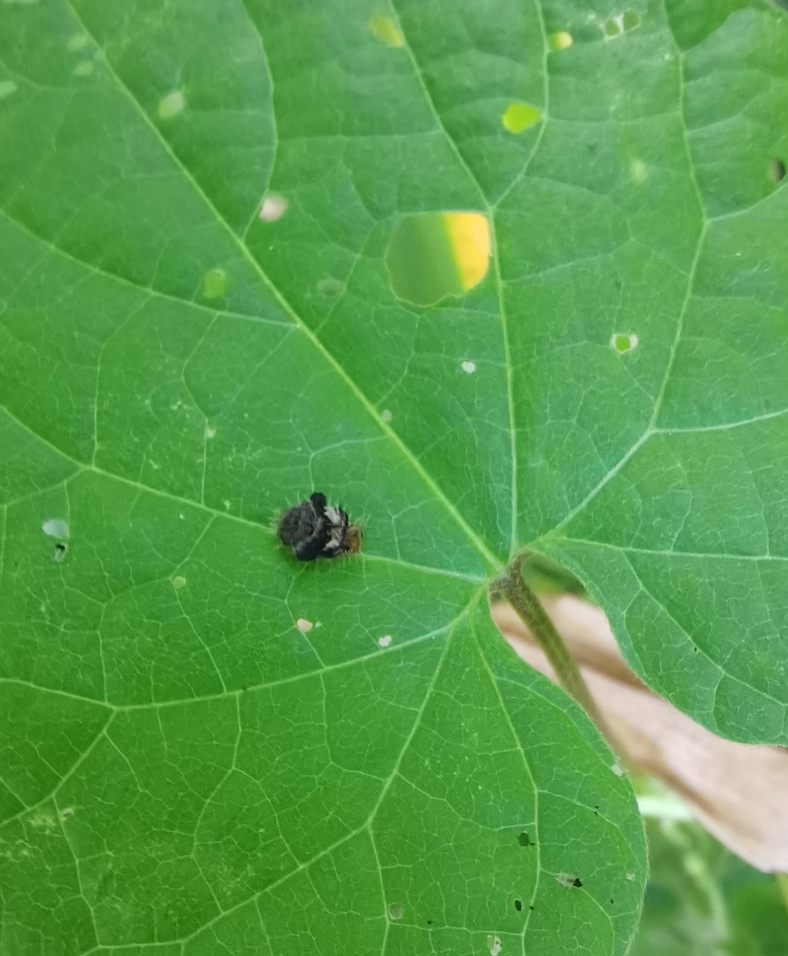
Larva;

**Figure 8c.**
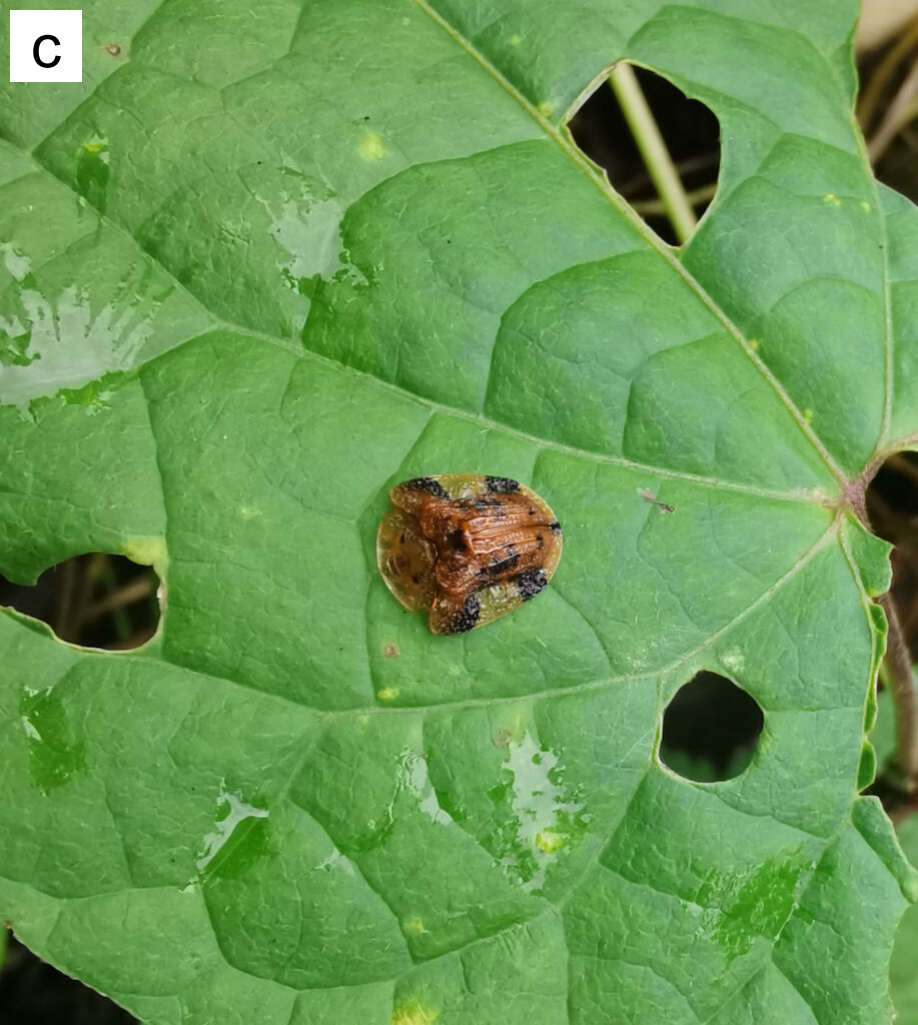
Adult;

**Figure 8d.**
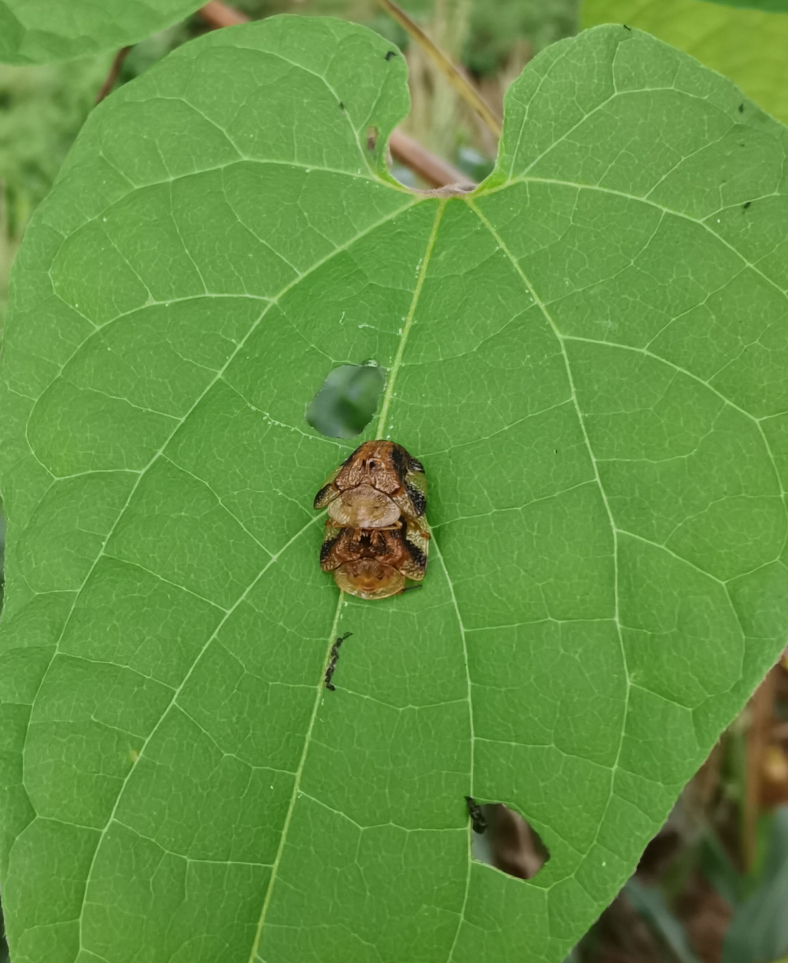
Adults copulating on the leaf upperside.

**Figure 9a.**
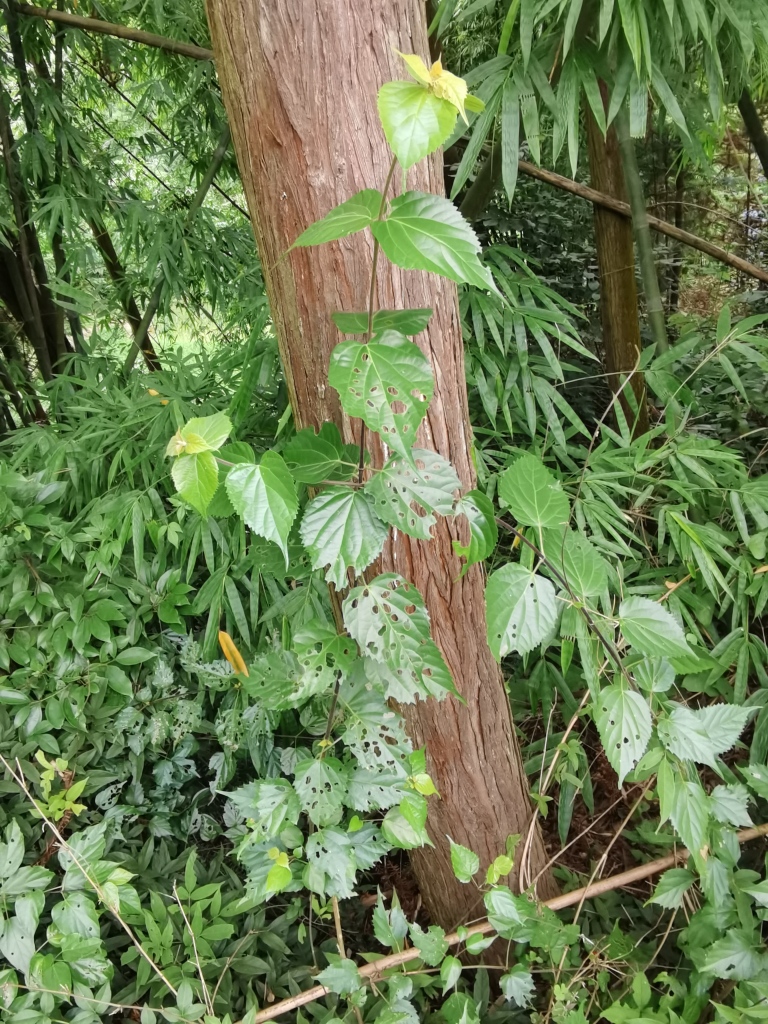
*Premnamicrophylla* Turcz. (Lamiaceae), the host plant for *B.pudica*;

**Figure 9b.**
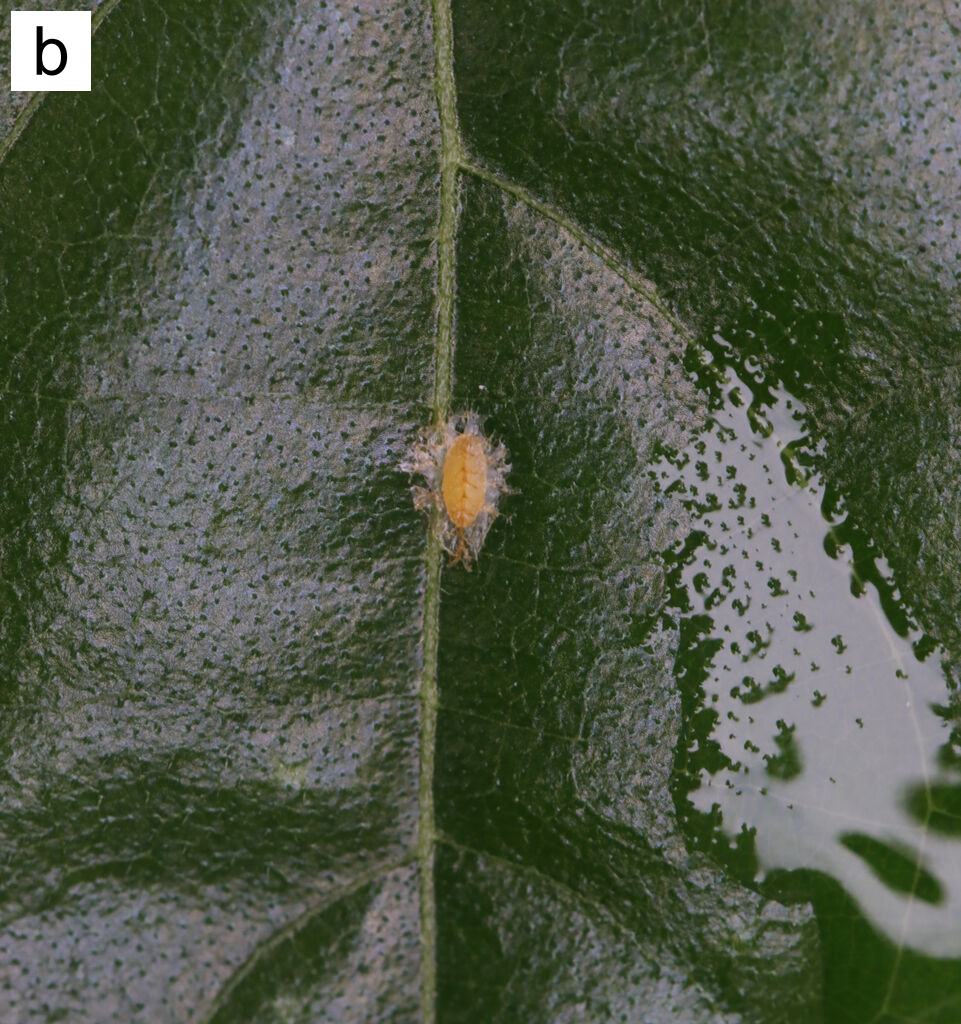
Egg;

**Figure 9c.**
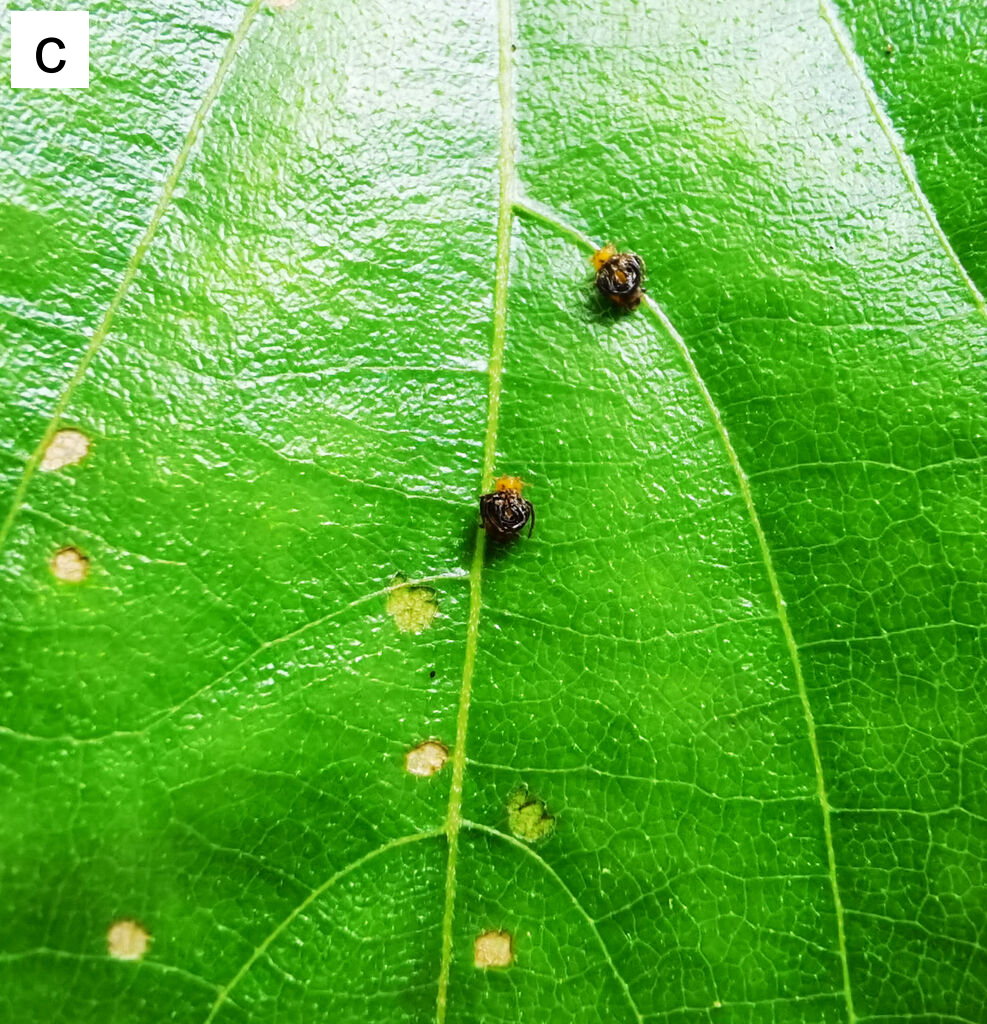
First instar larvae and their feeding pattern;

**Figure 9d.**
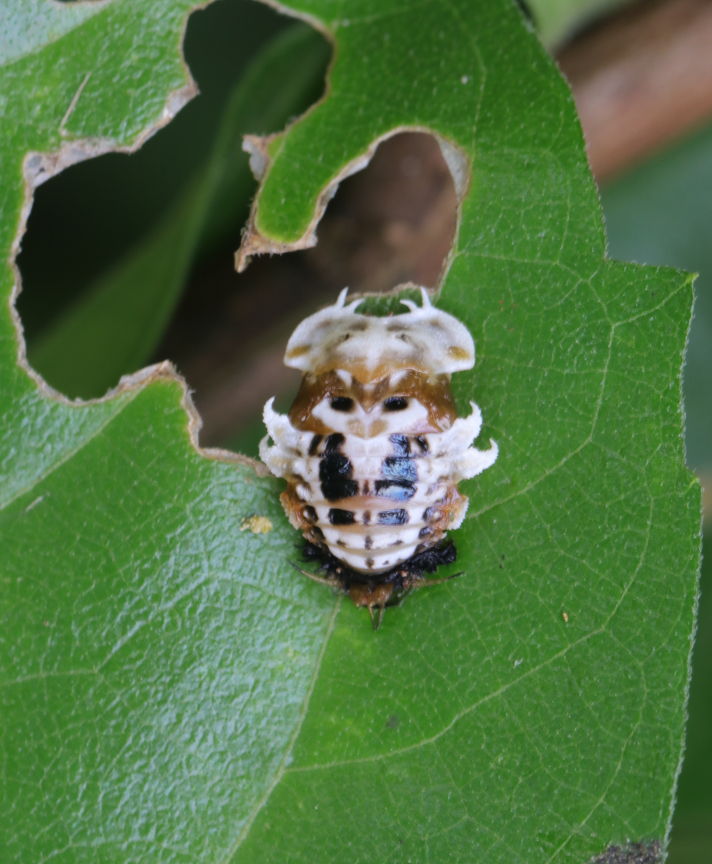
Mature larva;

**Figure 9e.**
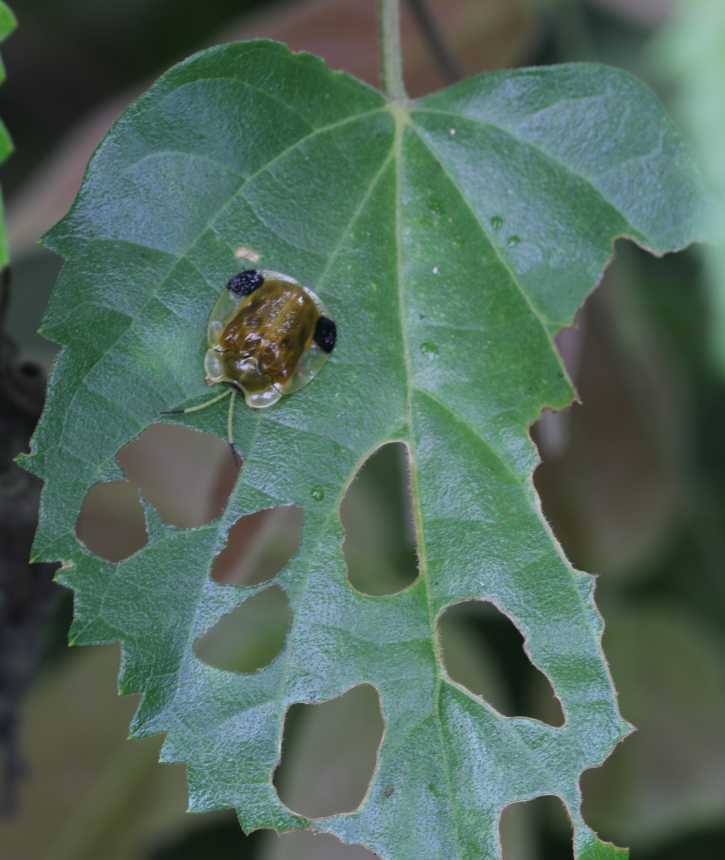
Adult and its feed pattern;

**Figure 9f.**
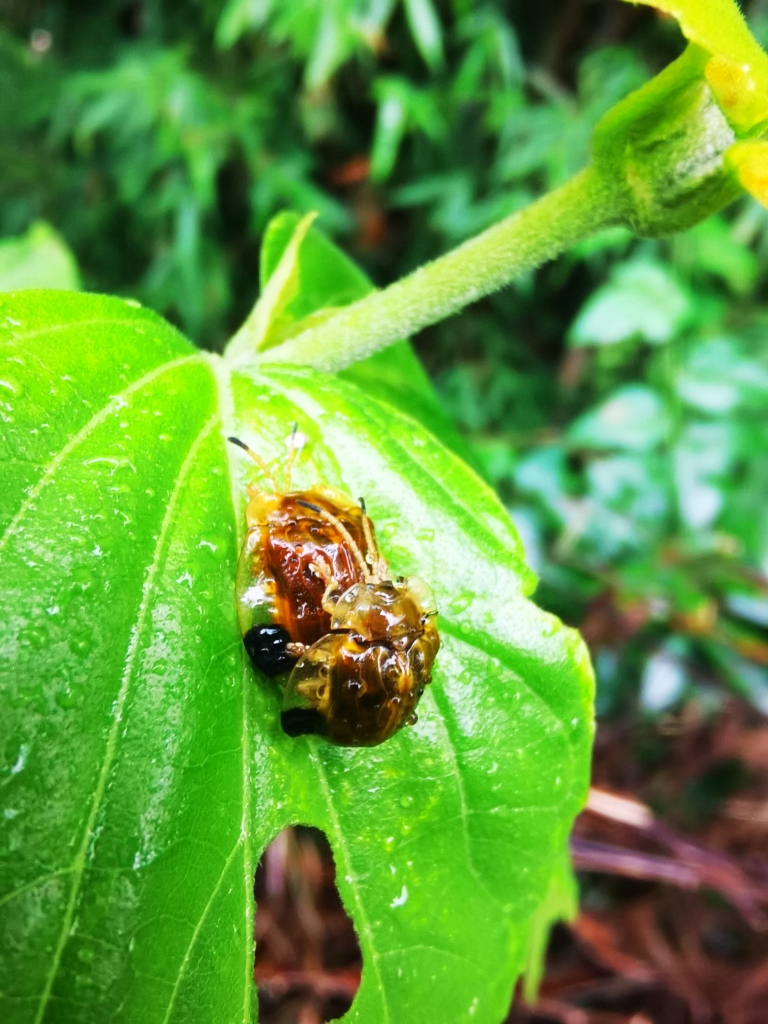
Adults copulating on the leaf upperside.

**Figure 10a.**
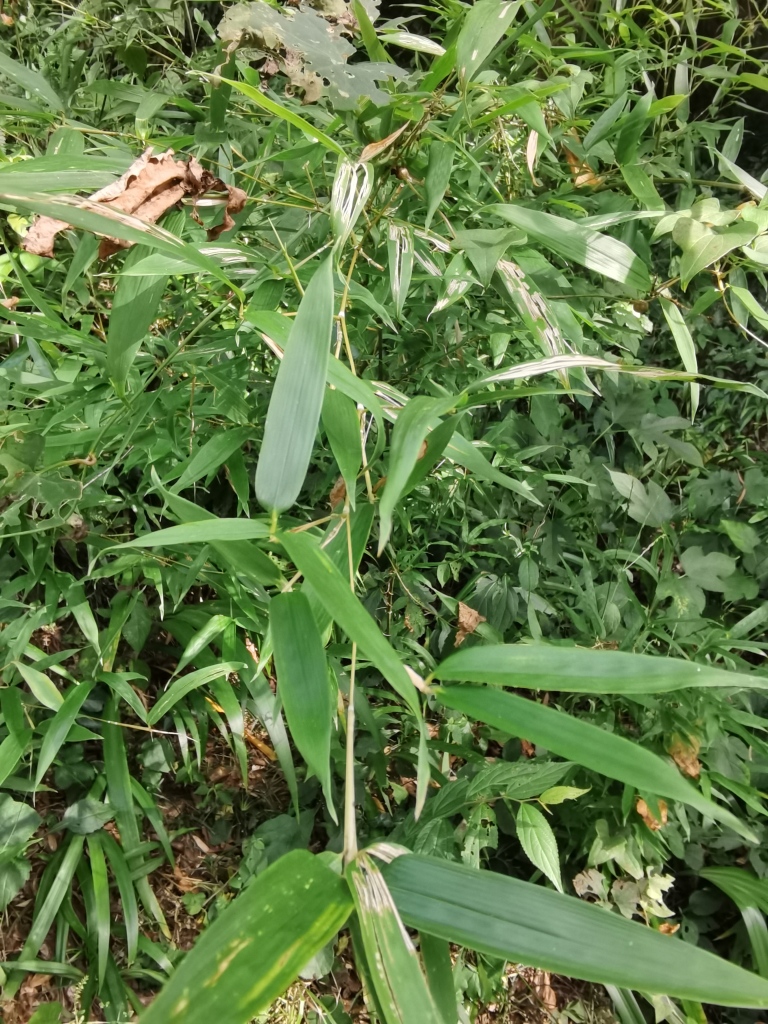
Phyllostachysnigravar.henonis (Mitford) Rendle (Poaceae), the host plant for *C.bowringii*;

**Figure 10b.**
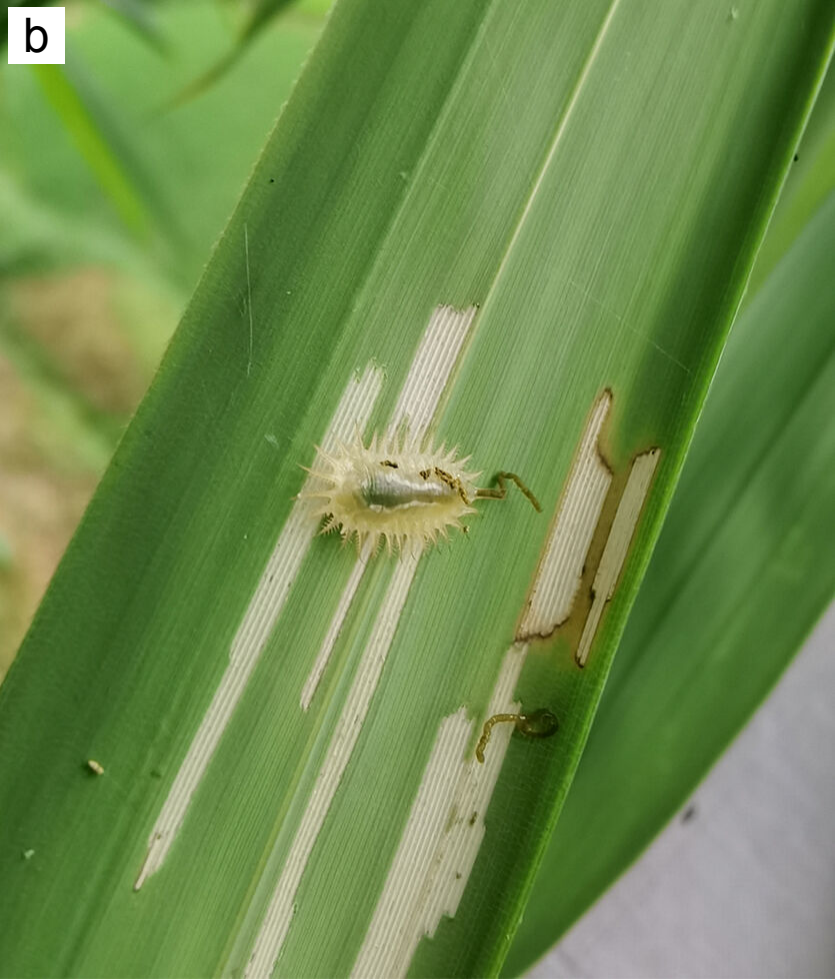
Larva and its feeding channels;

**Figure 10c.**
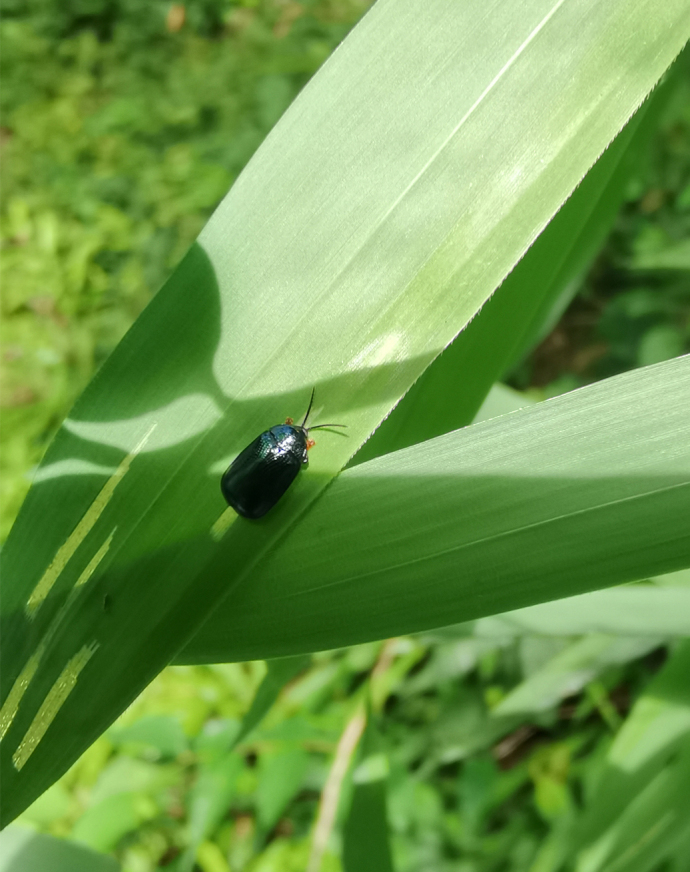
Adult and its feeding channels (lower side);

**Figure 10d.**
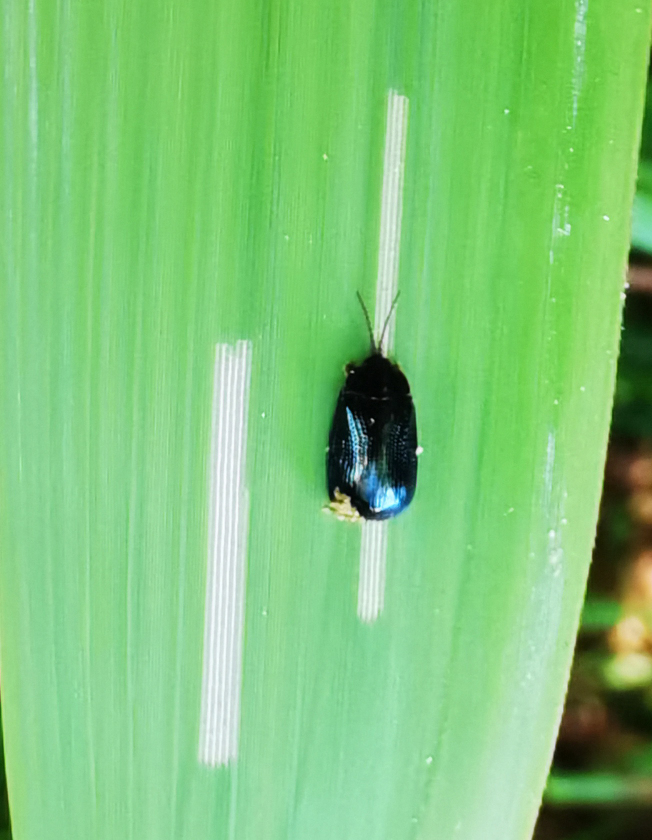
Adult and its feeding channels (upper side).

**Figure 11a.**
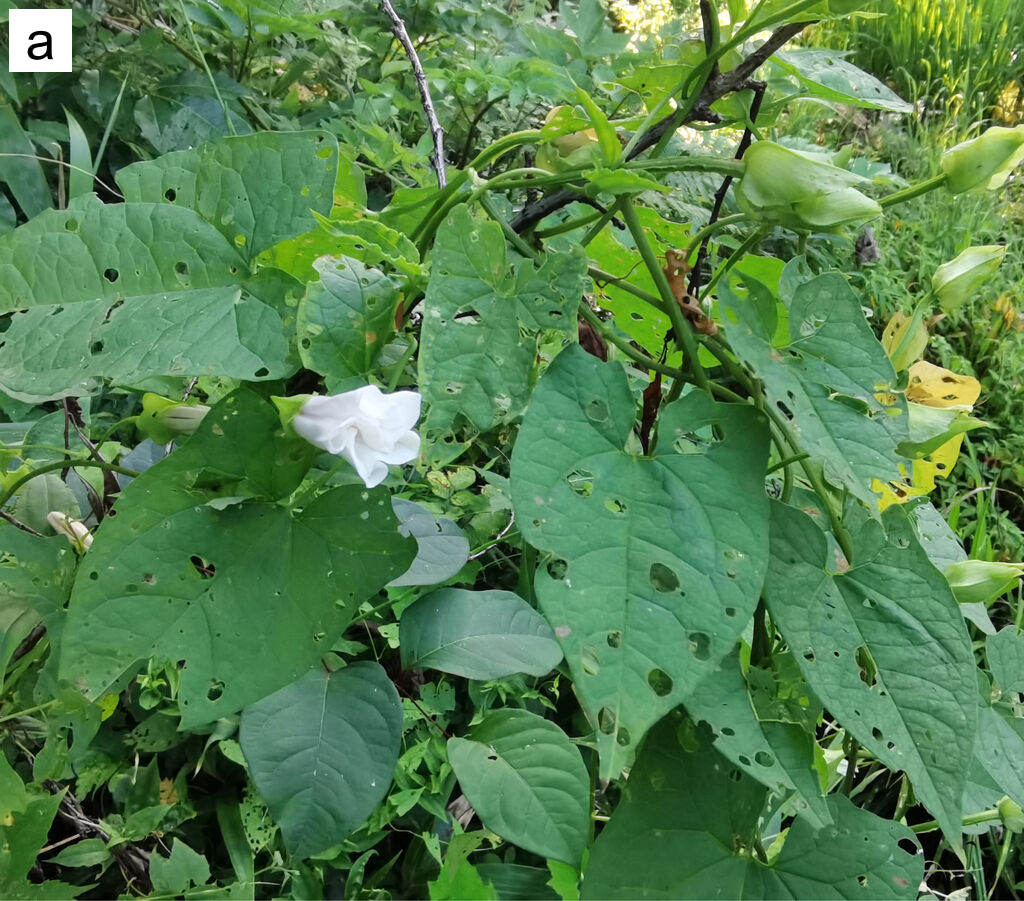
*Calystegiasepium* (L.) R.Br. (Convolvulaceae), the host plant for *G.spilota*;

**Figure 11b.**
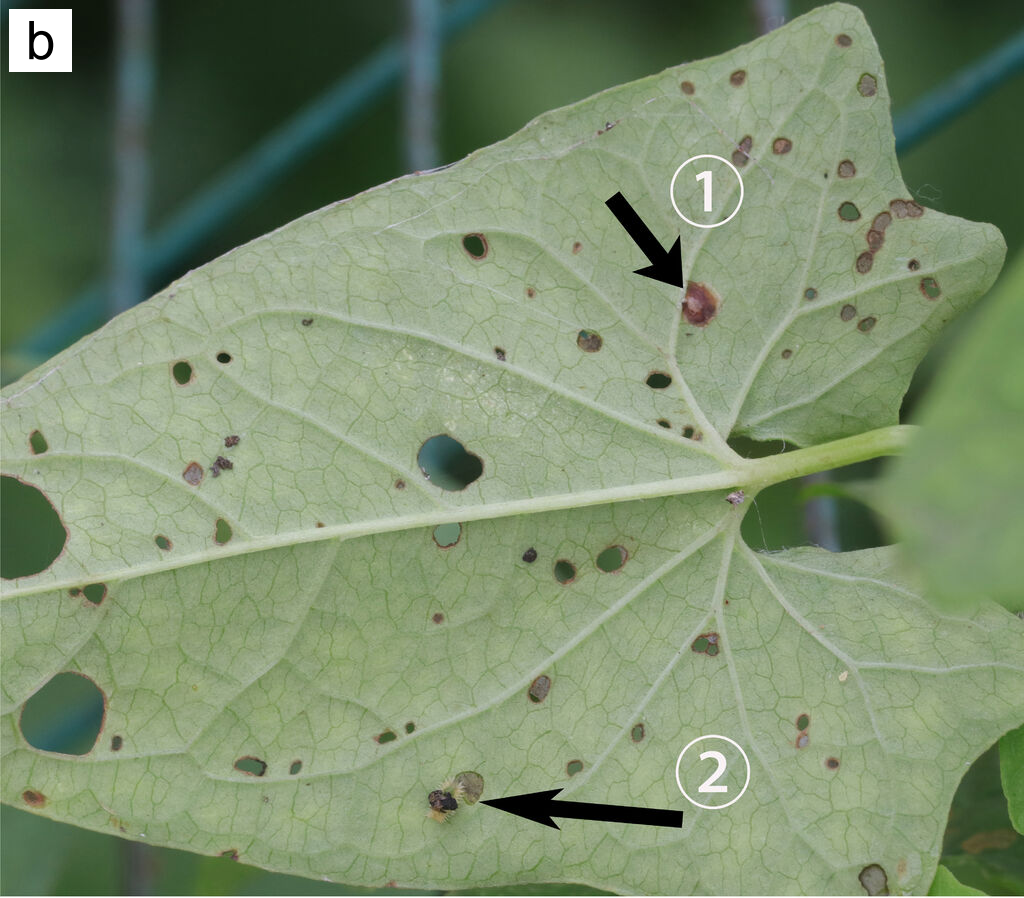
Egg and first larva;

**Figure 11c.**
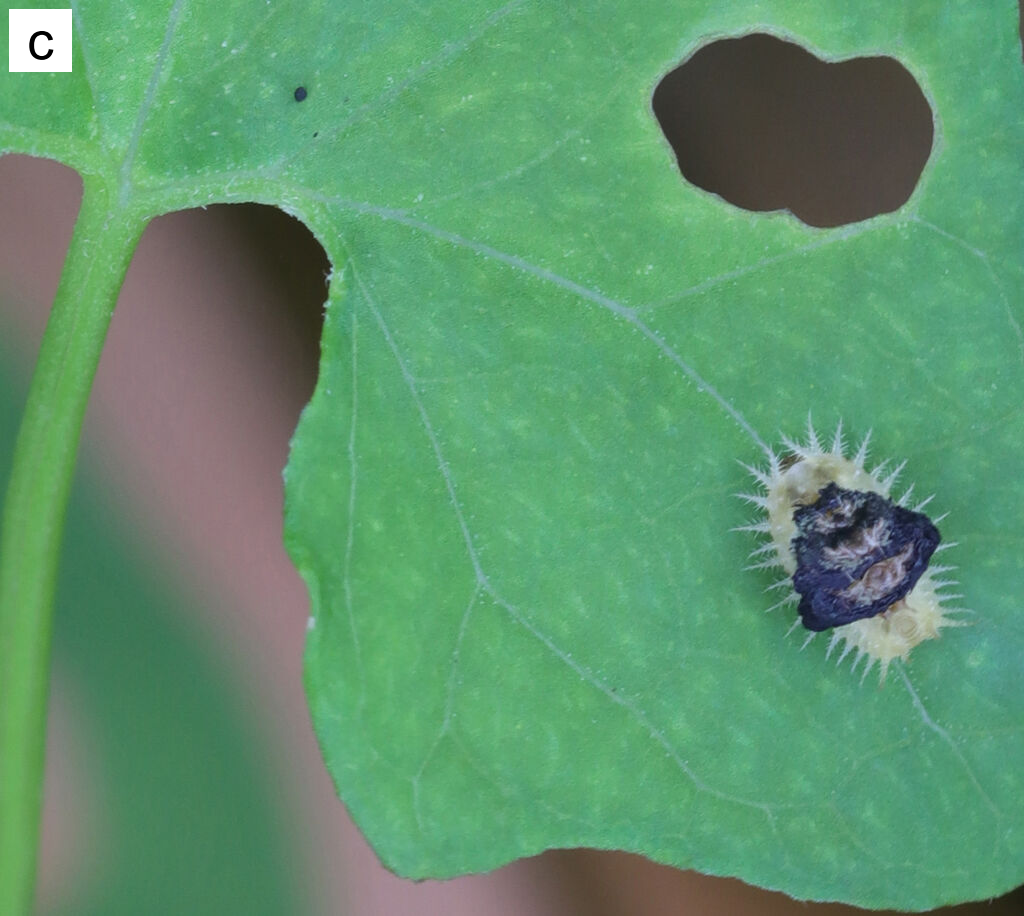
Larva;

**Figure 11d.**
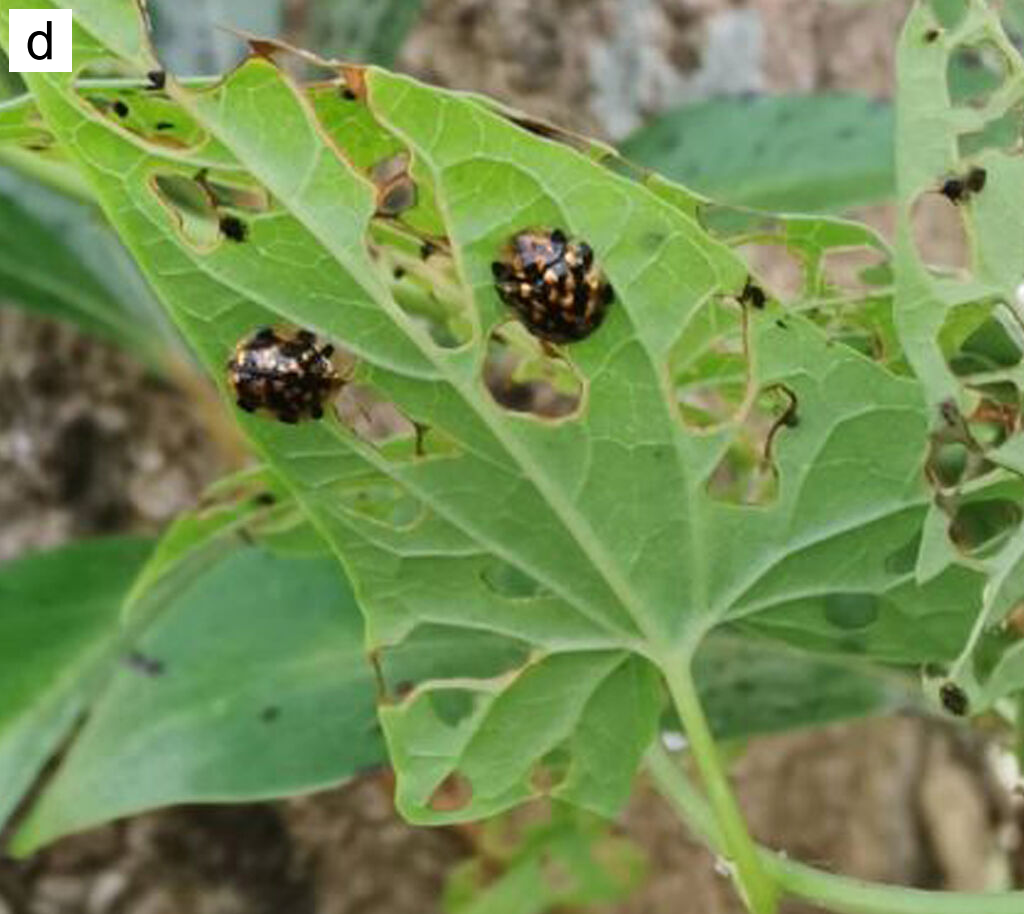
Adults and their feeding pattern.

**Figure 12a.**
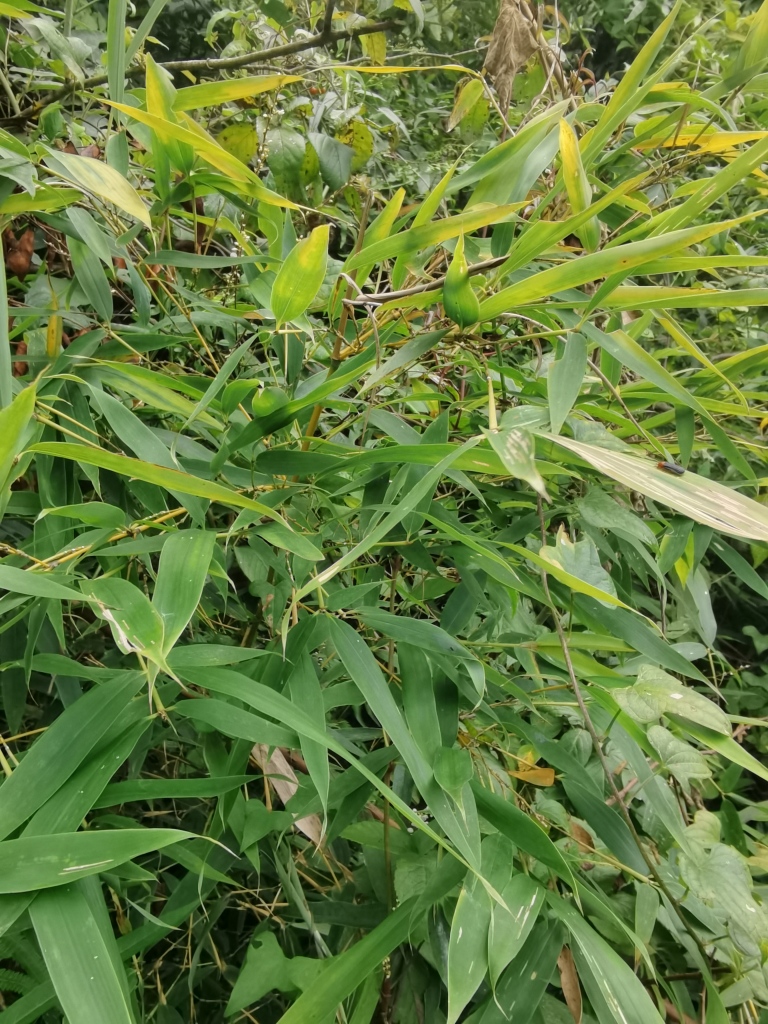
*Phyllostachysmannii* Gamble (Poaceae), the host plant for *A.chinensis*;

**Figure 12b.**
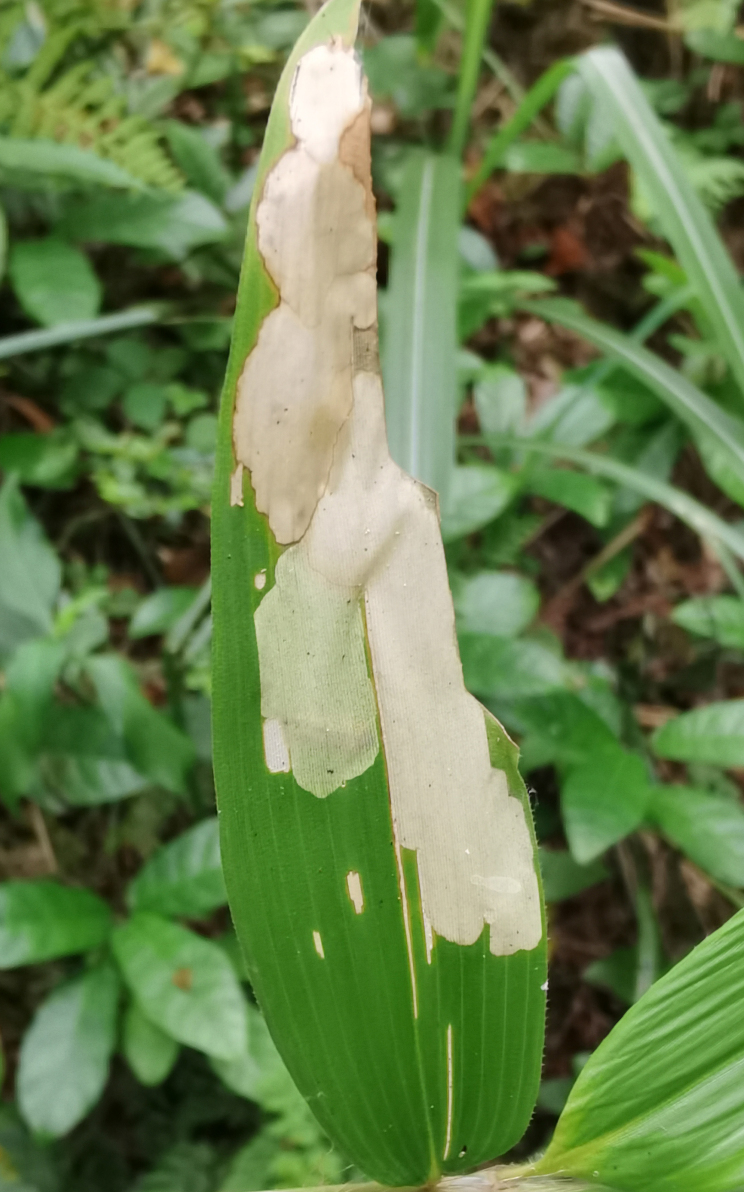
Larval mine;

**Figure 12c.**
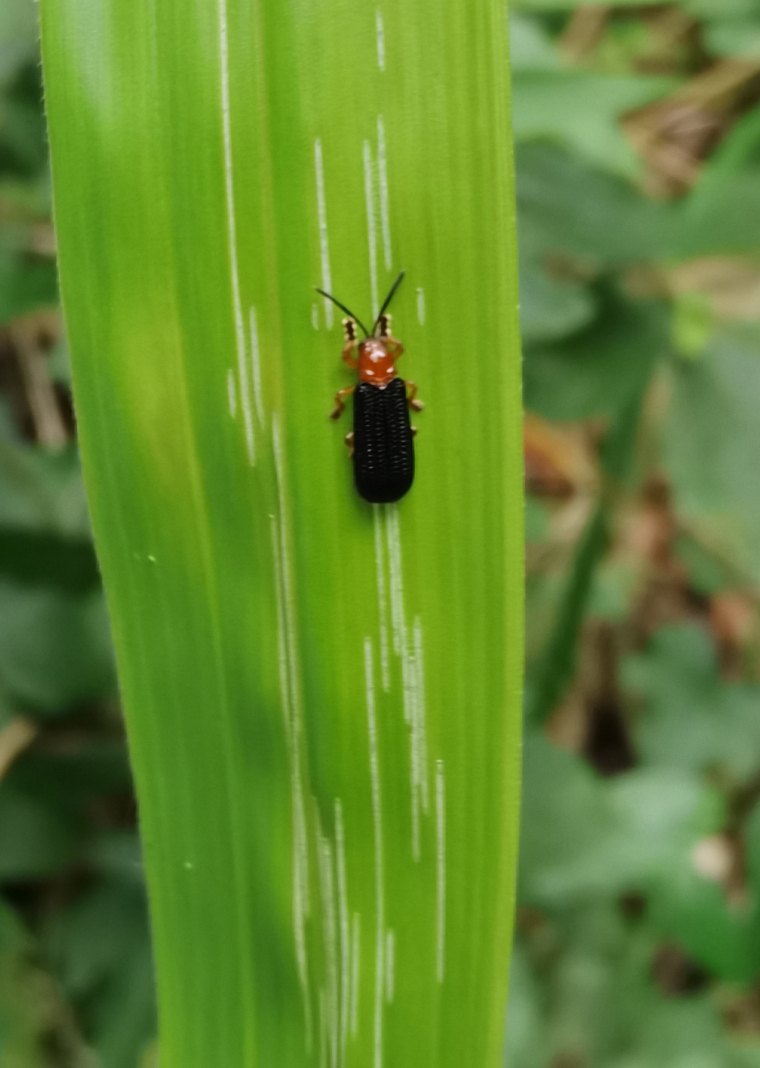
Adult and its feeding channels;

**Figure 12d.**
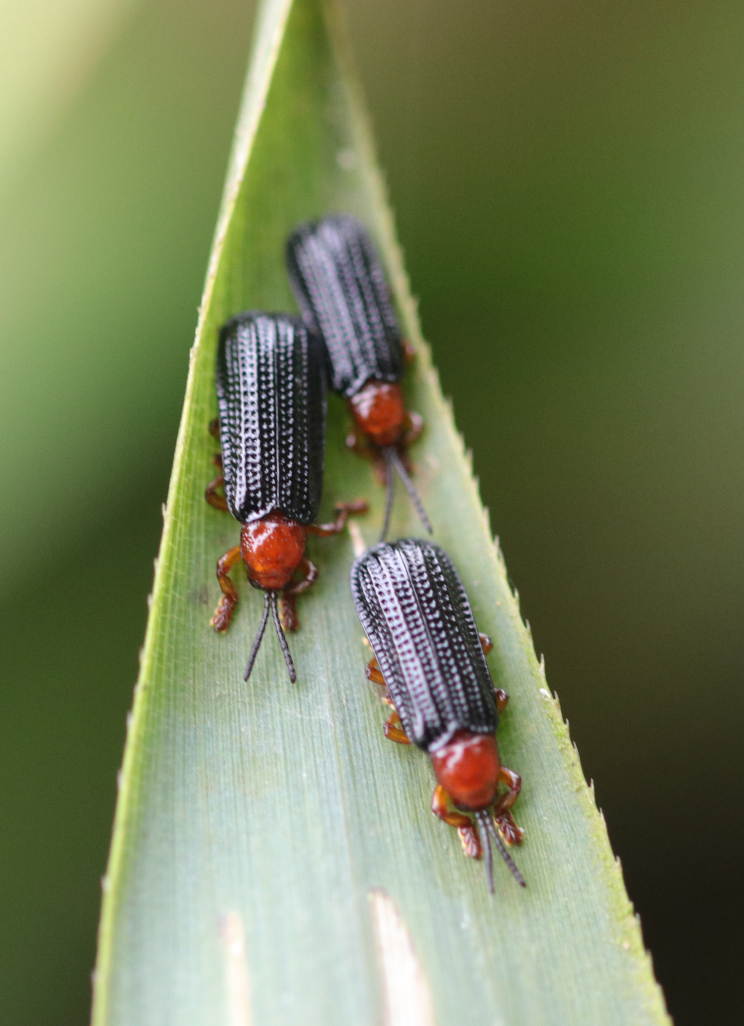
Adults overwinter in groups on the leaf of *Pleioblastusamarus*.

**Figure 13a.**
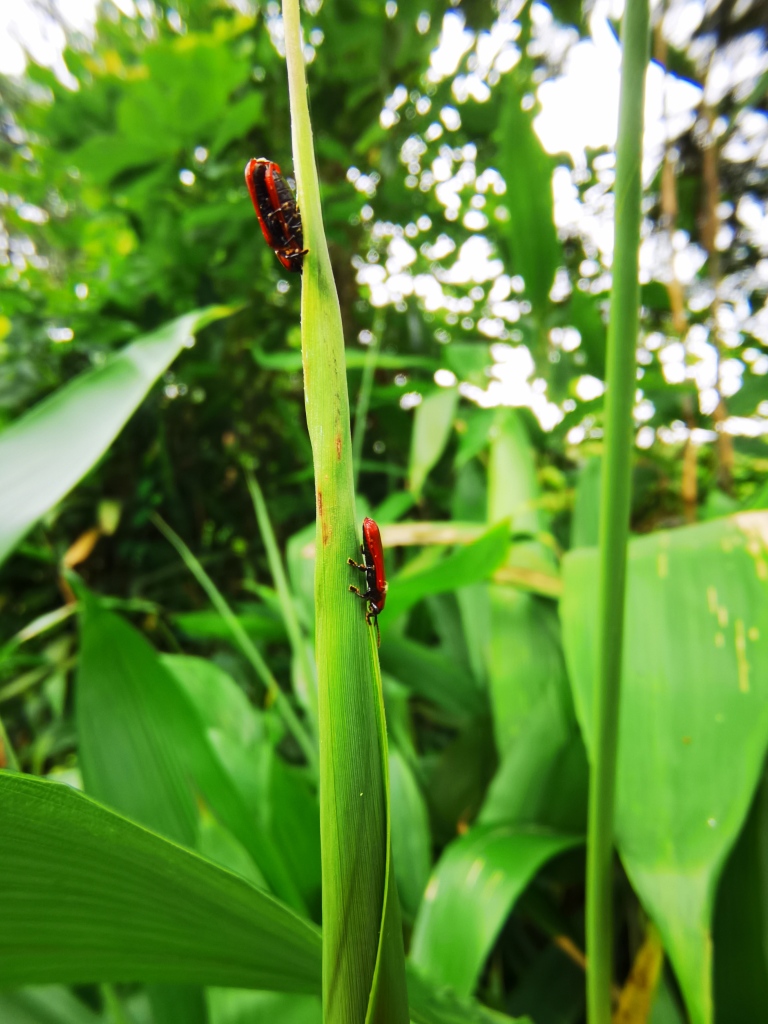
Adults copulating on the rolled leaf of *Indocalamustessellatus* (Munro) Keng f. (Poaceae);

**Figure 13b.**
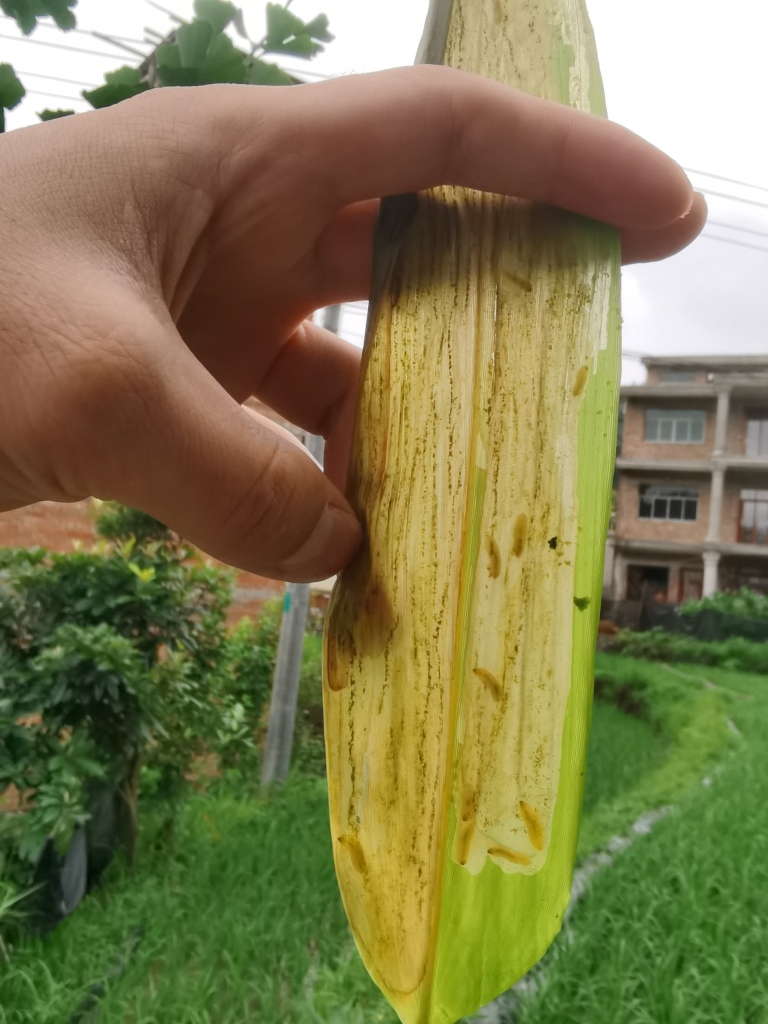
Larvae sharing a large mine in the same leaf;

**Figure 13c.**
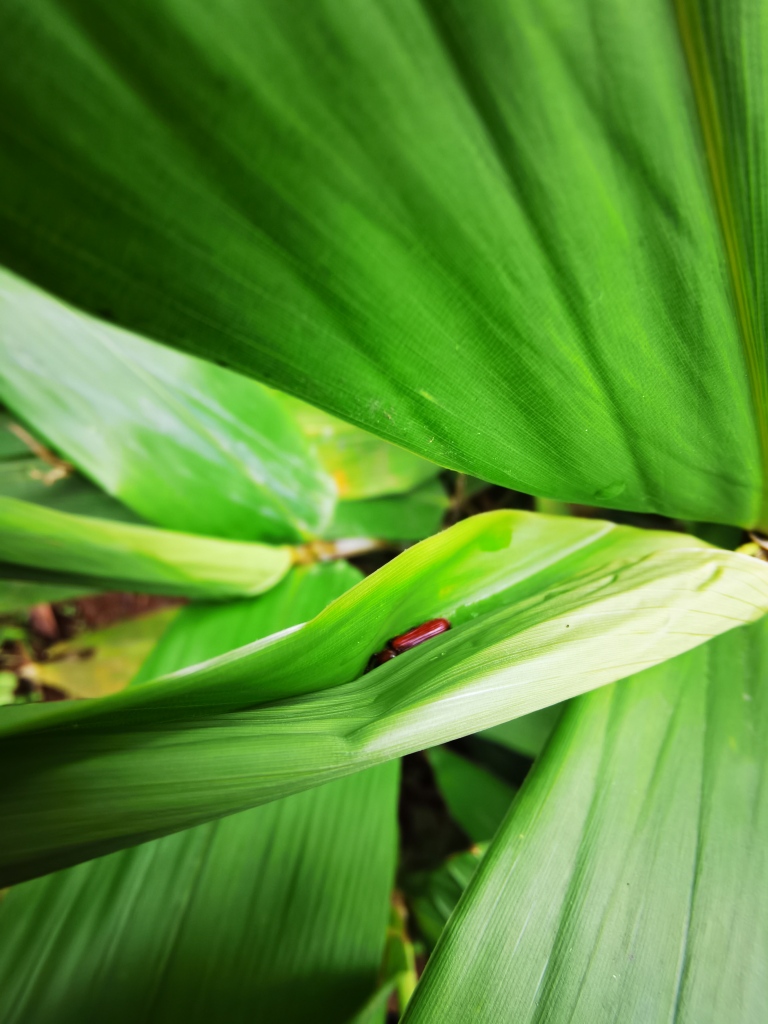
Adult feeding on the rolled leaf;

**Figure 13d.**
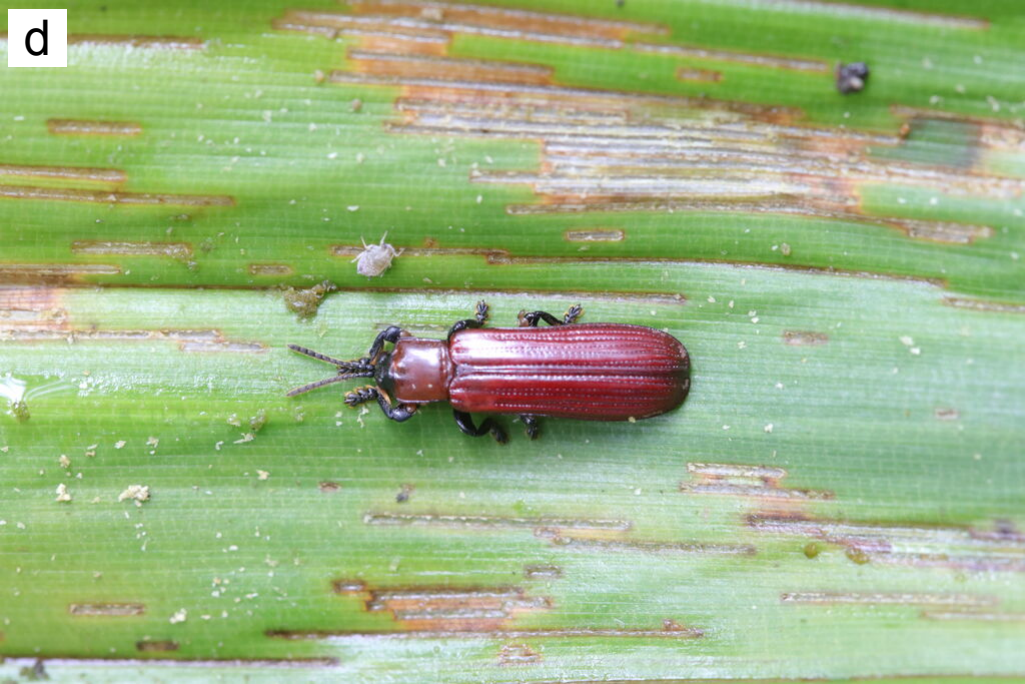
Adult and its feeding channels.

**Figure 14a.**
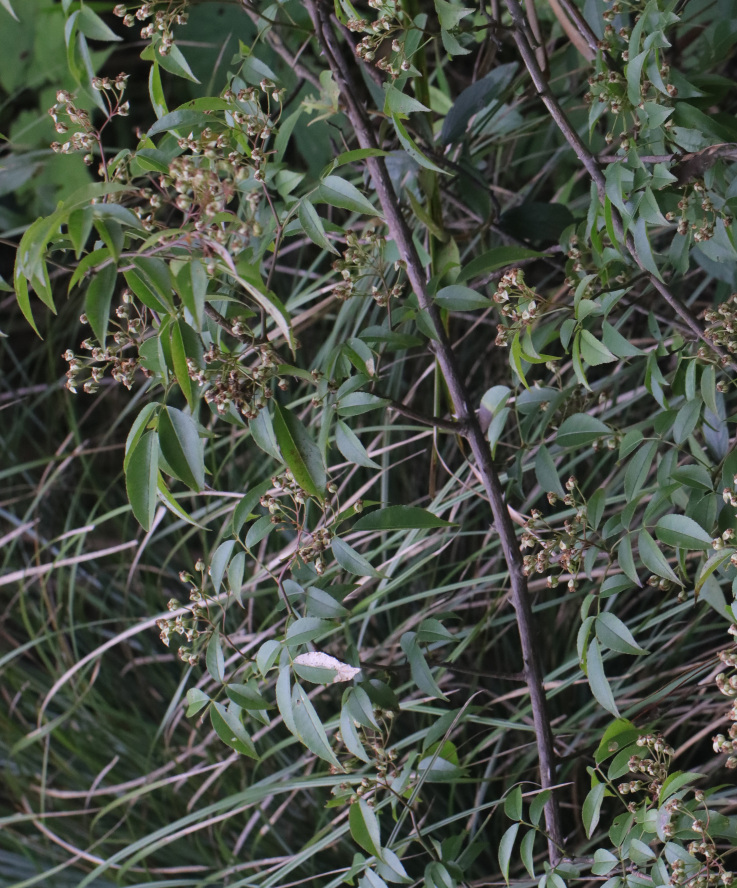
*Rosacymosa* Tratt. (Rosaceae), the host plant for *D.similis*;

**Figure 14b.**
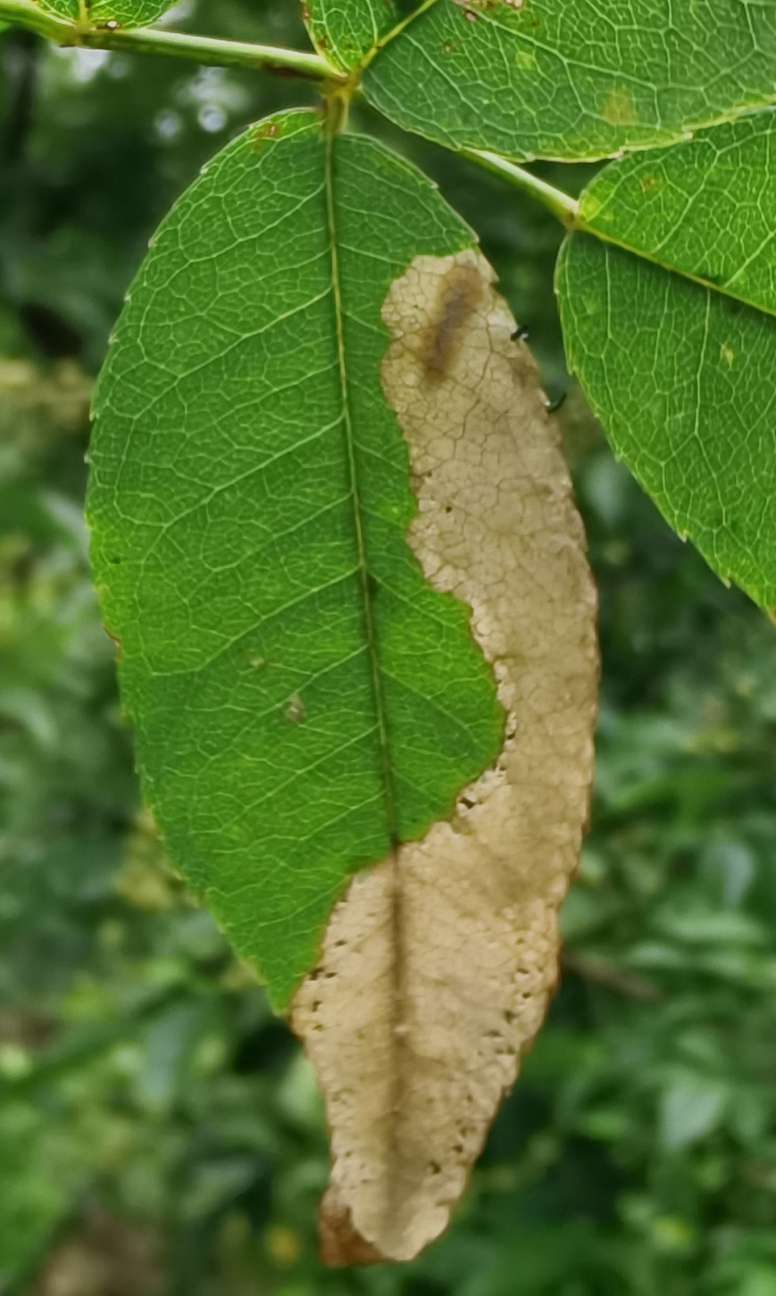
Larval mine;

**Figure 14c.**
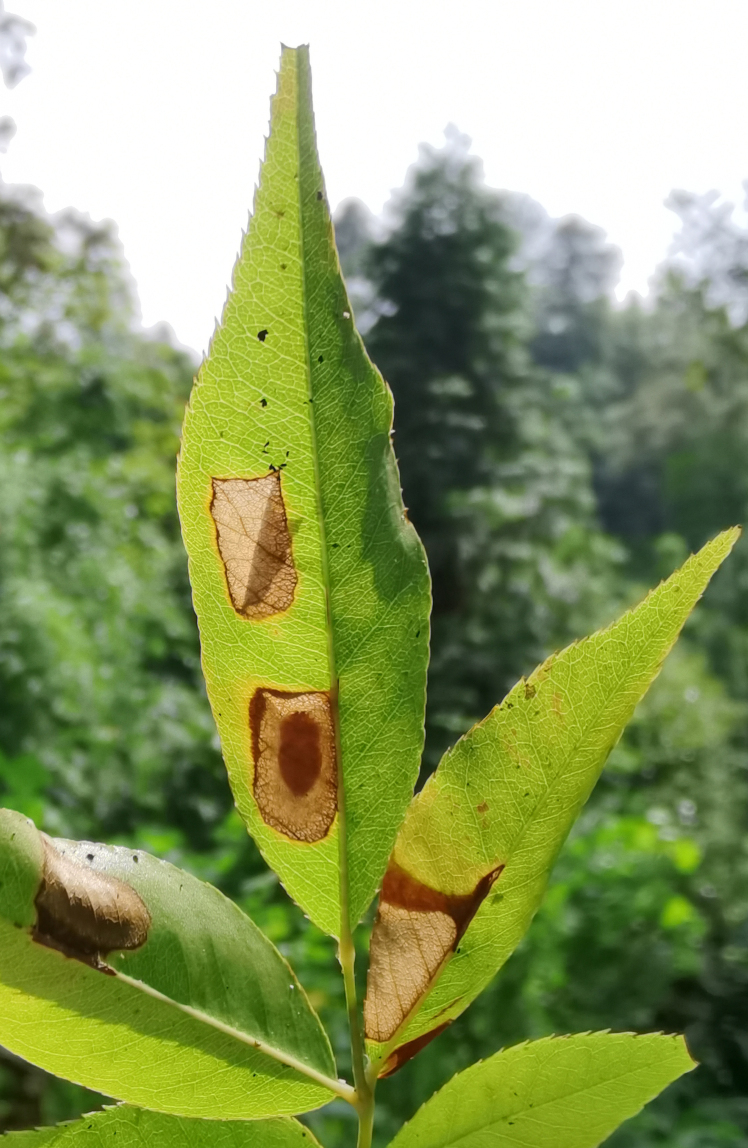
Pupal mine;

**Figure 14d.**
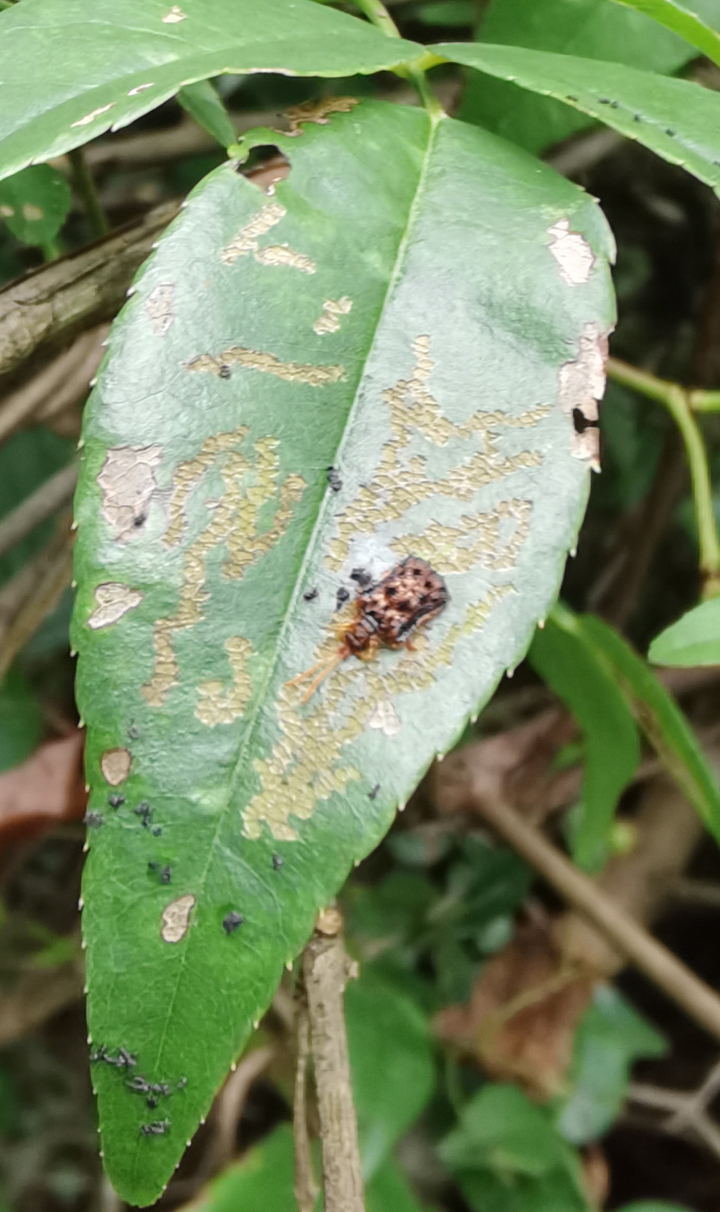
Adult and its feeding marks.

**Figure 15a.**
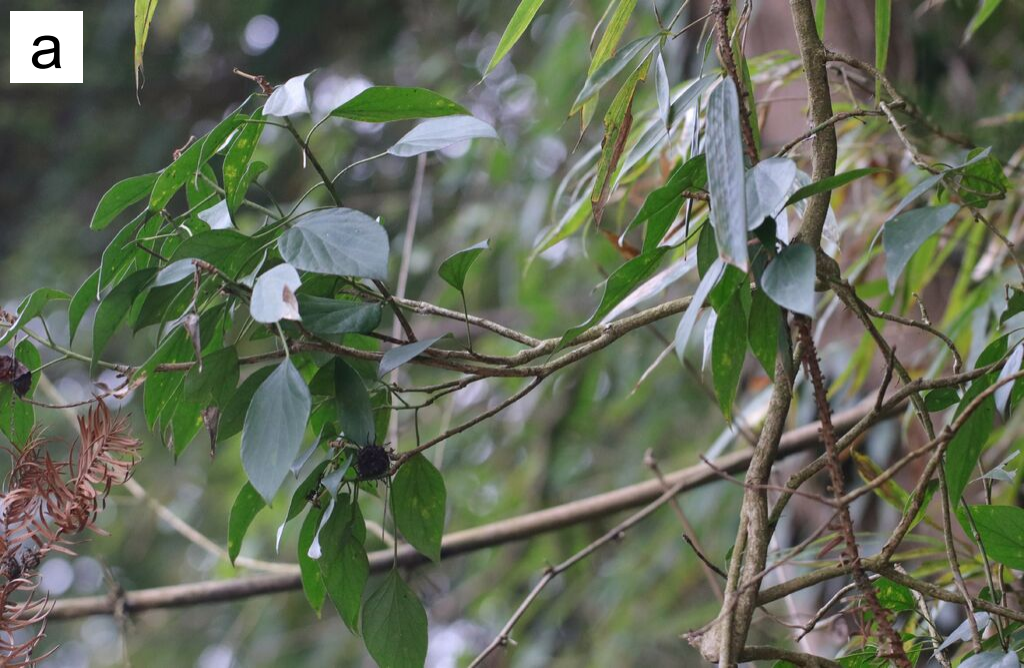
*Hederanepalensis* K.Koch (Araliaceae), the host plant for *D.gressitti*;

**Figure 15b.**
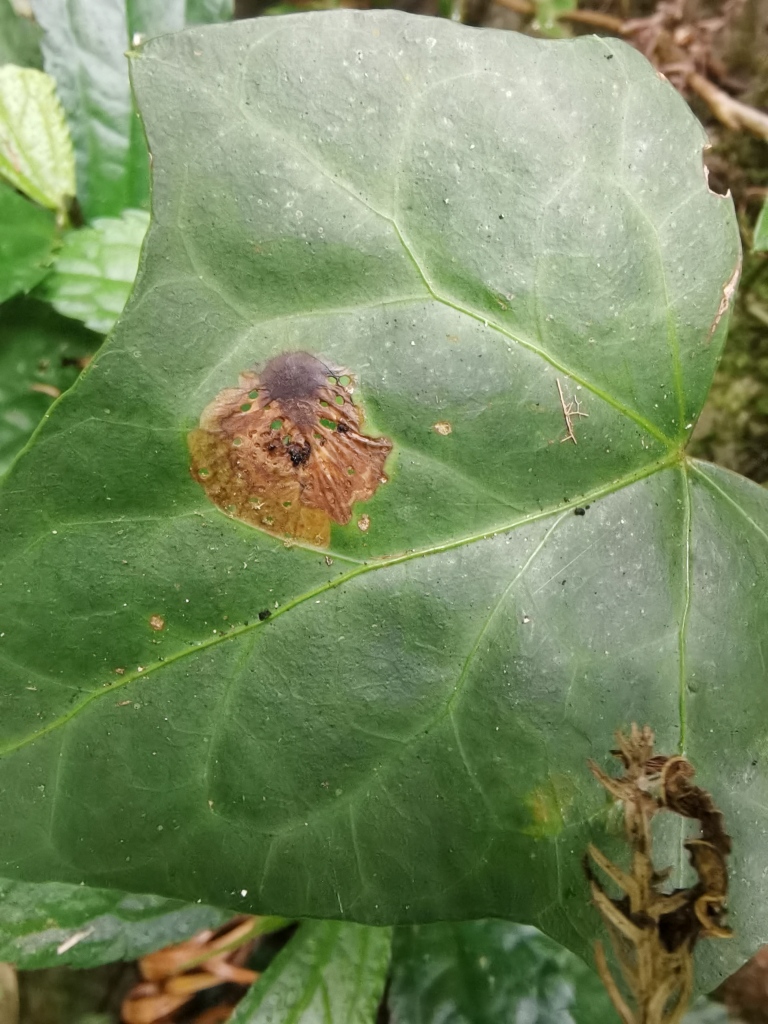
Larval mine;

**Figure 15c.**
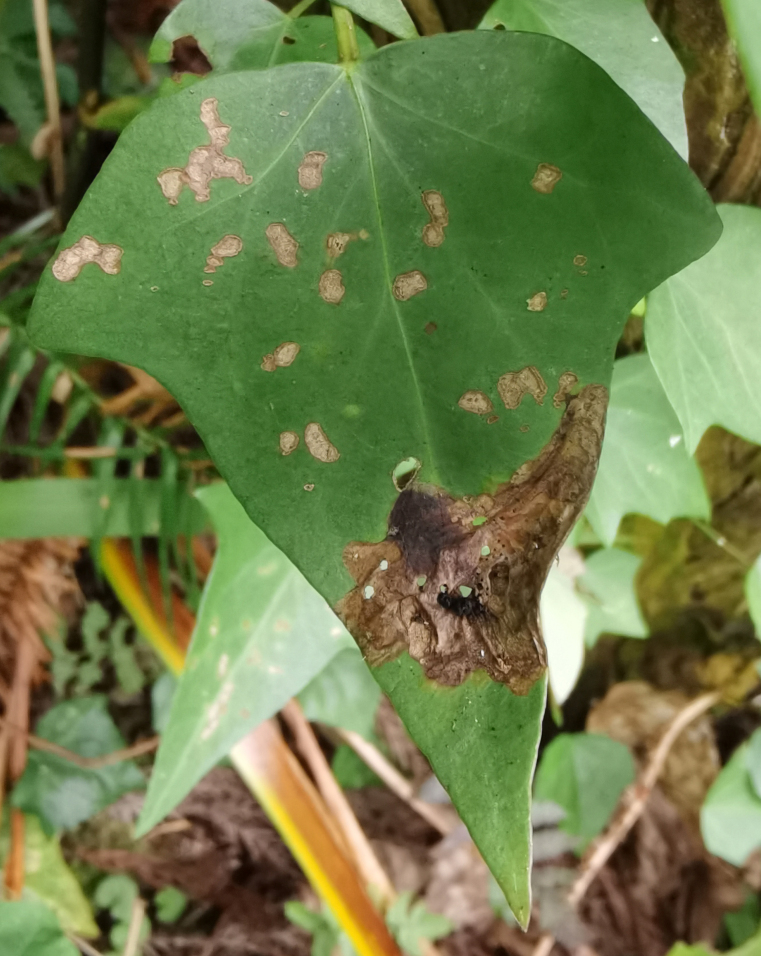
Mature larva mine and adult feeding marks;

**Figure 15d.**
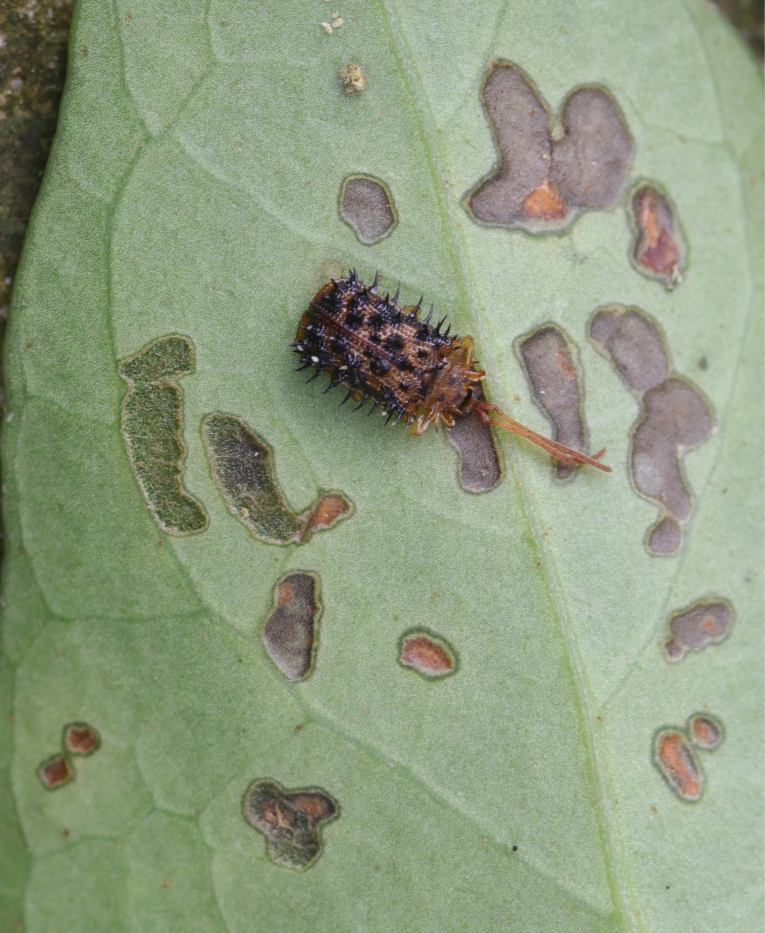
Adult and its feeding marks;

**Figure 15e.**
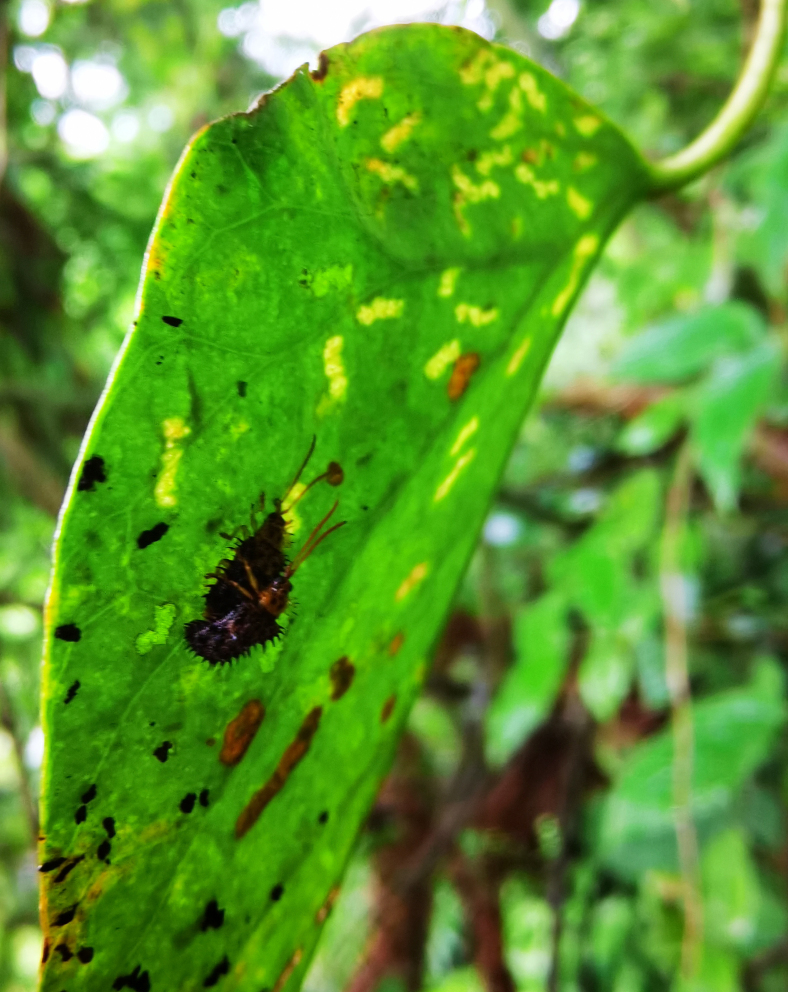
Adults copulating on the leaf underside.

**Figure 16a.**
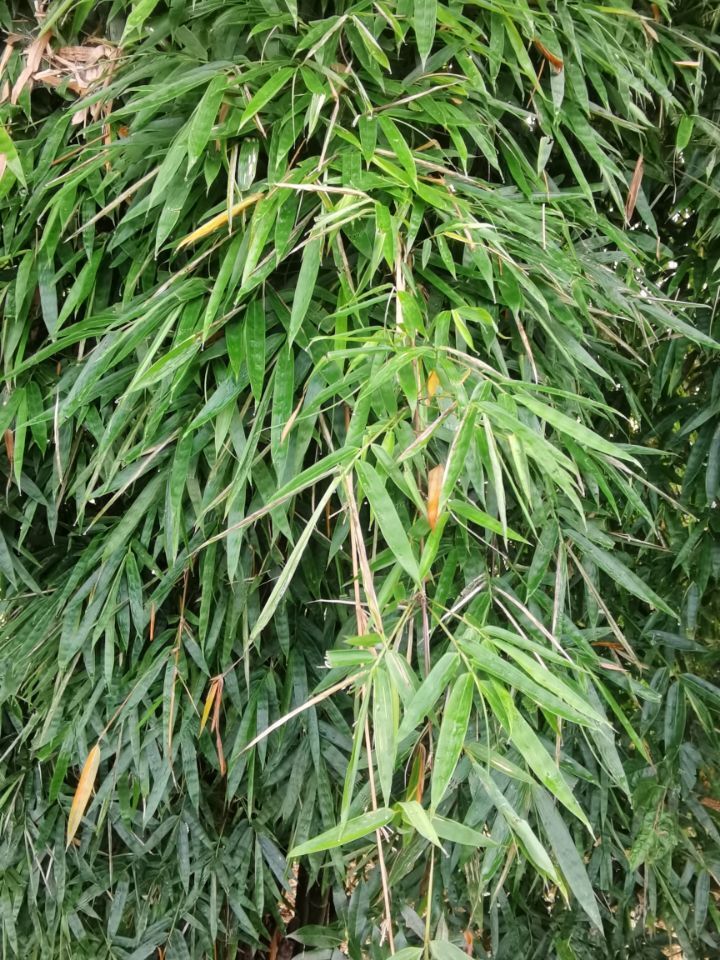
*Bambusablumeana* Schult.f. (Poaceae), the host plant for *L.pici*;

**Figure 16b.**
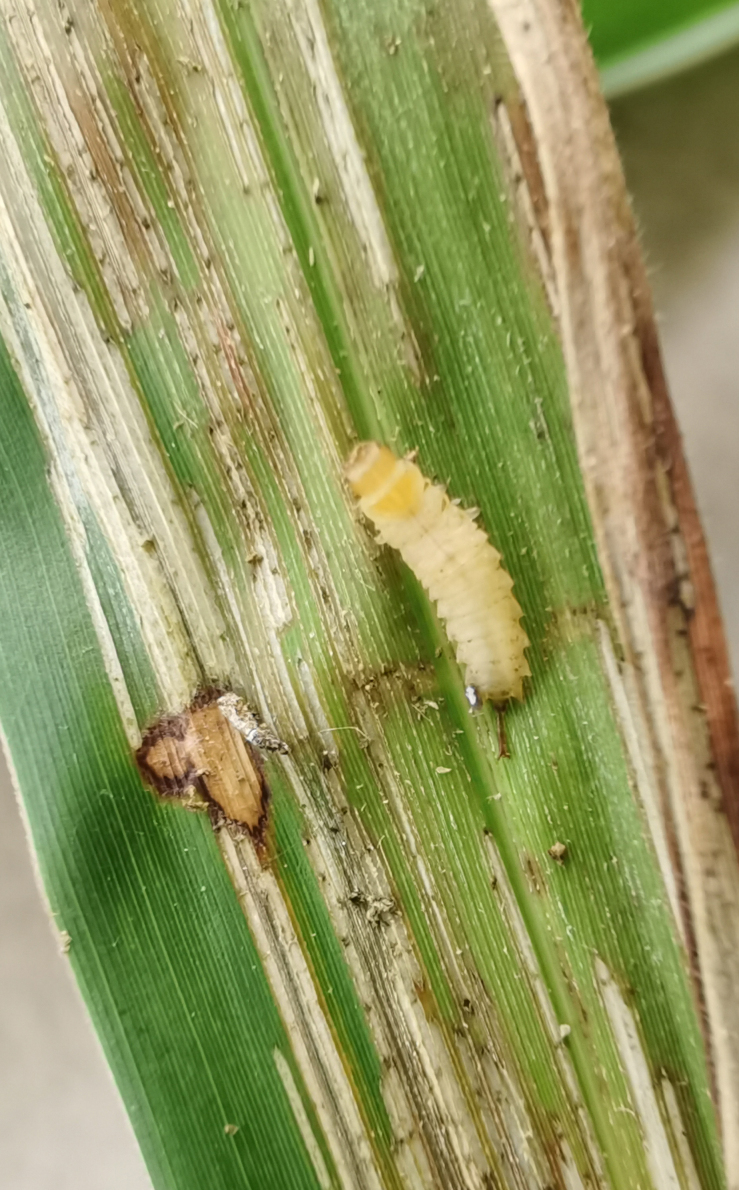
Larva;

**Figure 16c.**
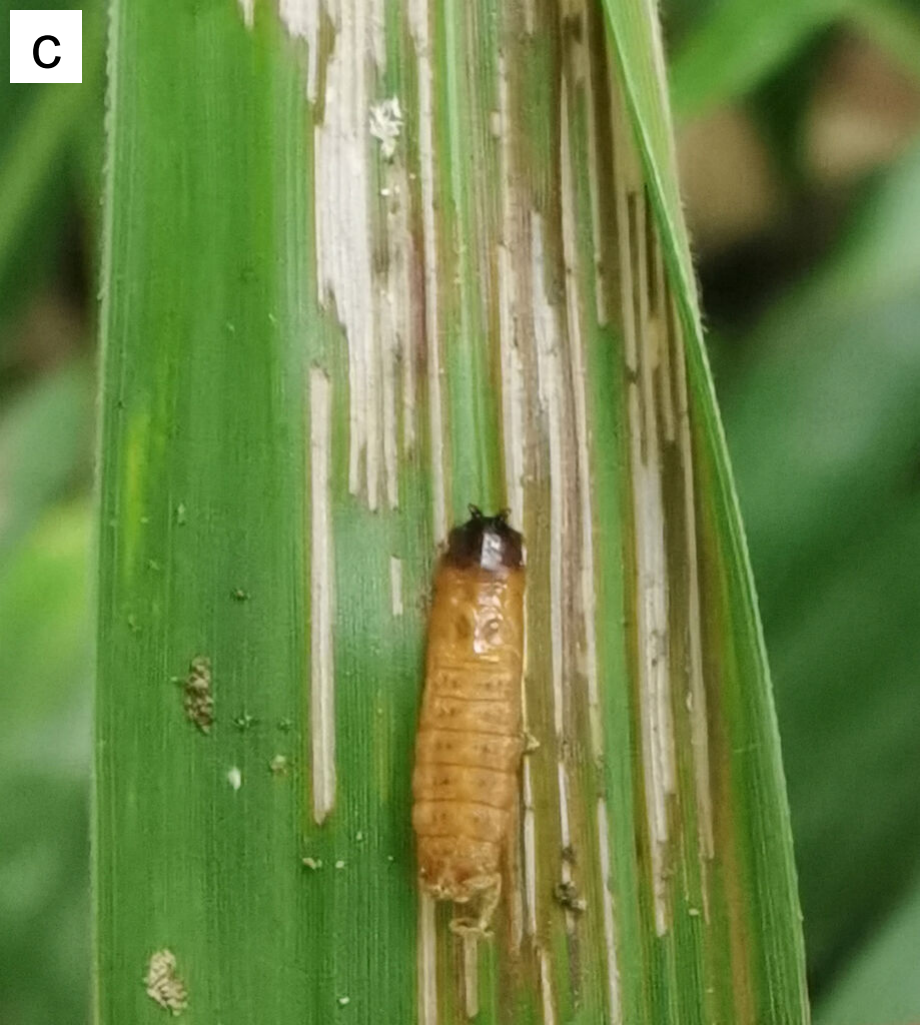
Pupa;

**Figure 16d.**
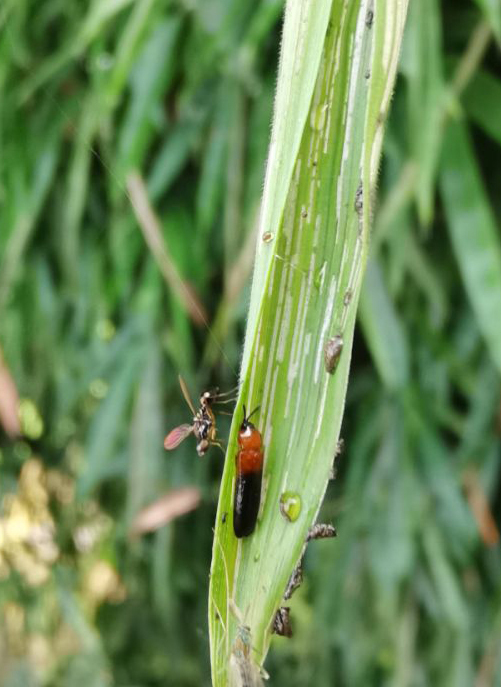
Adult and its feeding channels

**Figure 16e.**
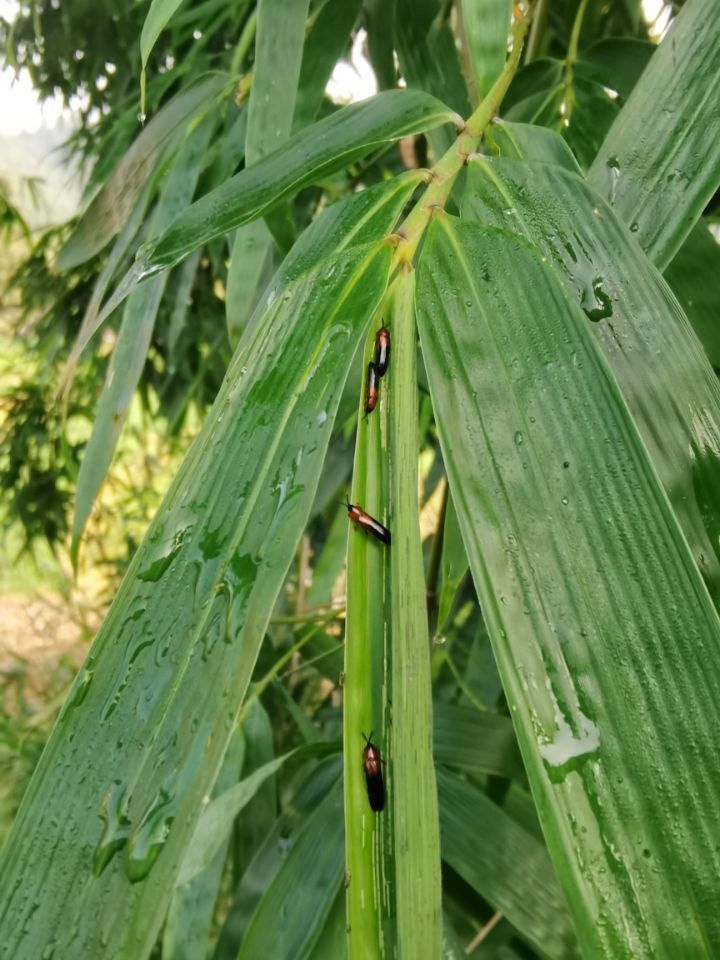
Adults feeding in groups inside the leaf roll.

**Table 1.** Cassidinae beetles and their host plants in Qiannan Prefecture. Note: "*" new record in Guizhou Province, "#" new record in Qiannan Prefecture, "**" new host record for the Cassidinae species, "-" unknown or unidentified plant.

**Cassidinae beetles**	**Host plant**
**Aspidimorphini**	
1 *Aspidimorpha* (*s. str.*) *difformis* (Motschulsky, 1860)^#^	*Dinetusracemosus* (Wallich) Sweet (Convolvulaceae)
2 *Aspidimorpha* (*s. str.*) *furcata* (Thunberg, 1789)	*Calystegiapubescens* Lindl. (Convolvulaceae) *Calystegiasepium* (L.) R. Br. (Convolvulaceae) Calystegiasilvaticasubsp.orientalis Brummitt (Convolvulaceae) *Dinetusracemosus* (Wallich) Sweet (Convolvulaceae) *Ipomoeabatatas* (L.) Lam. (Convolvulaceae) *Ipomoeapurpurea* (L.) Roth (Convolvulaceae)
3 Laccoptera (Laccopteroidea) nepalensis Boheman, 1855	*Calystegiasepium* (L.) R. Br. (Convolvulaceae) Calystegiasilvaticasubsp.orientalis Brummitt^**^ (Convolvulaceae) *Dinetusracemosus* (Wallich) Sweet (Convolvulaceae) *Ipomoeabatatas* (L.) Lam. (Convolvulaceae) *Ipomoeapurpurea* (L.) Roth (Convolvulaceae)
**Basiprionotini**	
4 *Basiprionotabisignata* (Boheman, 1862)	*Catalpafargesii* Bur.^**^ (Bignoniaceae) *Paulowniatomentosa* (Thunb.) Steud^**^ (Paulowniaceae) *Paulowniataiwaniana* T. W. Hu & H. J. Chang^**^ (Paulowniaceae)
5 *Basiprionotachinensis* (Fabricius, 1798)^#^	*Catalpafargesii* Bur.^**^ (Bignoniaceae) *Paulowniataiwaniana* T. W. Hu & H. J. Chang (Paulowniaceae)
6 *Basiprionotapudica* (Spaeth, 1925)^#^	*Premnamicrophylla* Turcz.^**^ (Lamiaceae)
**Callispini**	
7 *Callispabowringii* Baly, 1858	*Bambusablumeana* Schult.f.^**^ (Poaceae) *Indocalamustessellatus* (Munro) Keng f.^**^ (Poaceae) Phyllostachysnigravar.henonis (Mitford) Rendle^**^ (Poaceae) *Phyllostachyspropinqua* McClure^**^ (Poaceae)
8 *Callispabrettinghami* Baly, 1869^*^	*Saccharumarundinaceum* Retz.^**^ (Poaceae)
9 *Callispadimidiatipennis* Baly, 1858^*^	*Saccharumarundinaceum* Retz.^**^ (Poaceae)
10 *Callispadonckieri* Pic, 1924^*^	*Pleioblastusamarus* (Keng) Keng f.^**^ (Poaceae)
**Cassidini**	
11 *Cassidaaustralica* (Boheman, 1855)^*^	Calystegiasilvaticasubsp.orientalis Brummitt^**^ (Convolvulaceae) *Dinetusracemosus* (Wallich) Sweet^**^ (Convolvulaceae) *Ipomoeapurpurea* (L.) Roth^**^ (Convolvulaceae)
12 *Cassidacircumdata* Herbst, 1799	Calystegiasilvaticasubsp.orientalis Brummitt (Convolvulaceae) *Dinetusracemosus* (Wallich) Sweet^**^ (Convolvulaceae) *Ipomoeabatatas* (L.) Lam. (Convolvulaceae) *Ipomoeapurpurea* (L.) Roth (Convolvulaceae)
13 *Cassidajapana* Baly, 1874^#^	*Achyranthesbidentata* Blume^**^ (Amaranthaceae)
14 *Cassidarati* (Maulik, 1923)	*Clematisarmandii* Franch.^**^ (Ranunculaceae)
15 *Cassidaspaethiana* Gressitt, 1952^*^	*Achyranthesbidentata* Blume^**^ (Amaranthaceae)
16 *Cassidaversicolor* (Boheman, 1855)	*Photiniabodinieri* H. Lév. (Rosaceae) *Pyracanthafortuneana* (Maxim.) H.L. Li^**^ (Rosaceae) *Prunusconradinae* Koehne^**^ (Rosaceae) *Prunussalicina* Lindl.^**^ (Rosaceae) *Rubusswinhoei* Hance^**^ (Rosaceae)
17 *Cassidaverspertina* (Boheman, 1862)^#^	*Clematisarmandii* Franch.^**^ (Ranunculaceae)
18 *Glyphocassisspilota* (Gorham, 1885)^#^	*Calystegiasepium* (L.) R. Br.^**^ (Convolvulaceae) Calystegiasilvaticasubsp.orientalis Brummitt^**^ (Convolvulaceae) *Dinetusracemosus* (Wallich) Sweet^**^ (Convolvulaceae)
19 *Glyphocassistrilineata* (Hope, 1831)	*Ipomoeabatatas* (L.) Lam. (Convolvulaceae)
20 *Thlaspidabiramosa* (Boheman, 1855)	*Callicarpabodinieri* H. Lév. (Lamiaceae) *Callicarpakochiana* Makino (Lamiaceae) *Callicarpamacrophylla* Vahl (Lamiaceae)
21 *Thlaspidosomabrevis* Chen et Zia, 1964^*^	*Akebiatrifoliata* (Thunb.) Koidz.^**^ (Lardizabalaceae) *Stauntoniaangustifolia* (Wall.) R.Br. ex Wall.^**^ (Lardizabalaceae)
**Gonophorini**	
22 *Agonitachinensis* (Weise, 1922)^#^	*Bambusablumeana* Schult.f.^**^ (Poaceae) *Bambusavariostriata* (W. T. Lin) L. C. Chia & H. L. Fung^**^ (Poaceae) *Indocalamustessellatus* (Munro) Keng f.^**^ (Poaceae) *Phyllostachysmannii* Gamble^**^ (Poaceae) Phyllostachysnigravar.henonis (Mitford) Rendle^**^ (Poaceae) *Phyllostachyspropinqua* McClure^**^ (Poaceae) *Pleioblastusamarus* (Keng) Keng f.^**^ (Poaceae)
23 *Agonitafoveicollis* (Chen et T'an, 1962)^*^	*Cyperus* sp. (Cyperaceae)
24 *Downesiaatrata* Baly, 1869^*^	*Indosasalipoensis* C.D.Chu & K.M.Lan^**^ (Poaceae) *Pleioblastusamarus* (Keng) Keng f.^**^ (Poaceae)
26 *Downesiagracilis* Uhmann, 1954^*^	*Indosasalipoensis* C.D.Chu & K.M.Lan^**^ (Poaceae)
27 *Downesiajavana* Weise, 1922^*^	*Pleioblastusamarus* (Keng) Keng f.^**^ (Poaceae)
25 *Downesiaruficolor* Pic, 1924^*^	*Indosasalipoensis* C. D. Chu et K. M. Lan^**^ (Poaceae) *Indocalamustessellatus* (Munro) Keng f. (Poaceae)
28 *Downesiastrandi* Uhmann, 1943^*^	*Indocalamustessellatus* (Munro) Keng f.^**^ (Poaceae)
29 *Downesiatarsata* Baly, 1869^*^	*Pleioblastusamarus* (Keng) Keng f.^**^ (Poaceae)
30 *Downesiavandykei* Gressitt, 1939^*^	*Indocalamustessellatus* (Munro) Keng f. (Poaceae)
31 *Downesia* sp.1^*^	*Indocalamustessellatus* (Munro) Keng f. (Poaceae)
32 *Downesia* sp.2^*^	*Pleioblastusamarus* (Keng) Keng f. (Poaceae)
33 *Klitispamutilata* Chen et Sun, 1964^*^	*Miscanthusfloridulus* Warb. ex K.Schum. & Lauterb. (Poaceae)
**Hispini**	
34 *Dactylispaangulosa* (Solsky, 1871)^#^	*Asterageratoides* Turcz.^**^ (Asteraceae) *Artemisiaargyi* H. Lév. & Vaniot^**^ (Asteraceae) *Artemisiaverlotiorum* Lamotte^**^ (Asteraceae)
35 *Dactylispabalyi* (Gestro, 1890)^*^	-
36 *Dactylispacervicornis* Gressitt, 1950^#^	*Aidiacanthioides* (Champ. ex Benth.) Masam^**^ (Rubiaceae)
37 *Dactylispachinensis* Weise, 1905^#^	*Rubusalceifolius* Poir. (Rosaceae) *Rubusamphidasys* Focke (Rosaceae) *Rubusbuergeri* Miq.^**^ (Rosaceae) *Rubusichangensis* Hemsl. & Kuntze^**^ (Rosaceae) *Rubusinnominatus* S. Moore^**^ (Rosaceae) *Rubusmalifolius* Focke^**^ (Rosaceae) *Rubussetchuenensis* Bureau & Franch.^**^ (Rosaceae) *Rubusswinhoei* Hance^**^ (Rosaceae) Rubusxanthoneurusvar.glandulosus T.T. Yu & L.T. Lu^**^ (Rosaceae) *Rubusxanthoneurus* Focke ex Diels^**^ (Rosaceae)
38 *Dactylispacrassicuspis* Gestro, 1906^#^	Corylusheterophyllavar.sutchuenensis Franch.^**^ (Betulaceae)
39 *Dactylispadelicatula* (Gestro, 1888)^*^	*Bambusablumeana* Schult.f.^**^ (Poaceae)
40 *Dactylispaexcisa* (Kraatz, 1879)^#^	*Pyracanthafortuneana* (Maxim.) H.L.Li^**^ (Rosaceae)
41 *Dactylispaferrugineonigra* Maulik, 1919^*^	*Aidiacanthioides* (Champ. ex Benth.) Masam.^**^ (Rubiaceae)
42 *Dactylispagressitti* Uhmann, 1954^*^	*Hederanepalensis* K.Koch^**^ (Araliaceae)
43 *Dactylispahigoniae* (Lewis, 1896)^#^	*Callicarpabodinieri* H.Lév.^**^ (Lamiaceae) *Callicarpamacrophylla* Vahl (Lamiaceae)
44 *Dactylispaintermedia* Chen et T’an, 1961^*^	*Rubuscorchorifolius* L.f.^**^ (Rosaceae) *Rubusmalifolius* Focke^**^ (Rosaceae)
45 *Dactylispaissiki* Chujo, 1938^*^	*Bambusablumeana* Schult.f.^**^ (Poaceae) *Bambusavariostriata* (W. T. Lin) L. C. Chia & H. L. Fung^**^ (Poaceae) Phyllostachysnigravar.henonis (Mitford) Rendle^**^ (Poaceae) *Phyllostachyspropinqua* McClure^**^ (Poaceae)
46 *Dactylispaklapperichi* Uhmann, 1954^*^	*Rubusmalifolius* Focke^**^ (Rosaceae) *Photiniabodinieri* H.Lév.^**^ (Rosaceae)
47 *Dactylispalongispina* Gressitt, 1938^#^	*Setariapalmifolia* Stapf (Poaceae) *Setariaplicata* T. Cooke^**^ (Poaceae)
48 *Dactylispamaculithorax* Gestro, 1906^#^	*Photiniabodinieri* H.Lév. (Rosaceae) *Pyracanthafortuneana* (Maxim.) H.L.Li^**^ (Rosaceae)
49 *Dactylispamixta* Kung et T’an, 1961^*^	-
50 *Dactylispanigrodiscalis* Gressitt, 1938^*^	*Uncariarhynchophylla* Miq. (Rubiaceae)
51 *Dactylispapaucispina* Gressitt, 1939^*^	*Callicarpamacrophylla* Vahl^**^ (Lamiaceae)
52 *Dactylispapici* Uhmann, 1934^*^	*Aidiacanthioides* (Champ. ex Benth.) Masam. (Rubiaceae)
53 *Dactylispapolita* Chen et T’an, 1961^*^	*Miscanthussinensis* Andersson^**^ (Poaceae) *Oplismenusundulatifolius* P.Beauv.^**^ (Poaceae)
54 *Dactylispapungens* (Boheman, 1859)^*^	*Rosacymosa* Tratt.^**^ (Rosaceae)
55 *Dactylispasauteri* Uhmann, 1927^#^	*Lophatherumgracile* Brongn. (Poaceae) *Miscanthussinensis* Anderss. (Poaceae) *Setariapalmifolia* Stapf^**^ (Poaceae)
56 *Dactylispasetifera* (Chapuis, 1877)	-
57 *Dactylispasimilis* Chen et T’an, 1985^*^	*Carpinusturczaninowii* Hance^**^ (Betulaceae) *Rosacymosa* Tratt.^**^ (Rosaceae)
58 *Dactylispasjoestedti* Uhmann, 1928^#^	Phyllostachysnigravar.henonis (Mitford) Rendle^**^ (Poaceae)
59 *Dactylispauhmanni* Gressitt, 1954^#^	*Rubusinnominatus* S. Moore^**^ (Rosaceae) *Rubuslambertianus* Ser.^**^ (Rosaceae)
60 *Dactylispa* sp.	*Bambusavariostriata* (W. T. Lin) L. C. Chia & H. L. Fung (Poaceae)
61 *Hispellinuscallicanthus* (Bates, 1866)	*Miscanthussinensis* Andersson^**^ (Poaceae)
62 Platypria (Platypria) acanthion Gestro, 1890^*^	*Phanerachampionii* Benth.^**^ (Fabaceae)
63 Platypria (Platypria) hystrix Fabricius, 1798^*^	Corylusheterophyllavar.sutchuenensis Franch.^**^ (Betulaceae)
64 *Rhadinosanigrocyanea* (Motschulsky, 1861)	*Miscanthussinensis* Andersson^**^ (Poaceae)
**Leptispini**	
65 *Leptispagodwini* Baly, 1869^*^	*Indocalamustessellatus* (Munro) Keng f.^**^ (Poaceae)
66 *Leptispalongipennis* (Gestro, 1890)^*^	*Bambusablumeana* Schult.f.^**^ (Poaceae)
67 *Leptispapici* Uhmann, 1958^*^	*Bambusablumeana* Schult.f.^**^ (Poaceae)
**Notosacanthini**	
68 *Notosacanthacastanea* (Spaeth, 1913)^*^	*Pittosporumillicioides* Mak.^**^ (Pittosporaceae)
69 *Notosacanthaginpinensis* Chen et Zia, 1961^*^	*Brideliabalansae* Tutcher^**^ (Phyllanthaceae)
